# THF‐solvated Heavy Alkali Metal Benzyl Compounds (Na, Rb, Cs): Defined Deprotonation Reagents for Alkali Metal Mediation Chemistry

**DOI:** 10.1002/chem.202103430

**Published:** 2021-10-25

**Authors:** Lukas Brieger, Christian Unkelbach, Carsten Strohmann

**Affiliations:** ^1^ Inorganic Chemistry TU Dortmund University Otto-Hahn-Str. 6/6a 44227 Dortmund Germany

**Keywords:** alkali metals, carbanions, hydroamination, main group elements, organometallic chemistry

## Abstract

The incorporation of heavy alkali metals into substrates is both challenging and essential for many reactions. Here, we report the formation of THF‐solvated alkali metal benzyl compounds [PhCH_2_M ⋅ (thf)_n_] (M=Na, Rb, Cs). The synthesis was carried out by deprotonation of toluene with the bimetallic mixture *n*‐butyllithium/alkali metal *tert*‐butoxide and selective crystallization from THF of the defined benzyl compounds. Insights into the molecular structure in the solid as well as in solution state are gained by single crystal X‐ray experiments and NMR spectroscopic studies. The compounds could be successfully used as alkali metal mediating reagents. The example of caesium showed the convenient use by deprotonating acidic C−H as well as N−H compounds to gain insight into the aminometalation using these reagents.

## Graphical abstract

**The selective formation** of THF‐solvated alkali metal benzyl compounds provides a decisive toolbox for alkali metal mediation chemistry and the introduction of the heavier congeners of lithium into small molecules. Crystal structures, NMR studies as well as quantum chemical calculations give detailed insights into these compounds. Experimental investigations towards deprotonations with application in aminometalation reactions are presented.

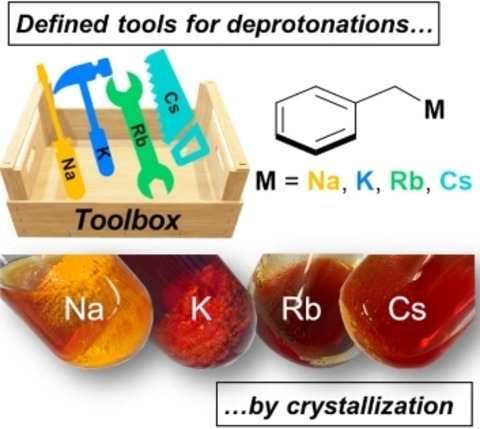

Interest in alkali metal mediated chemistry with the heavy alkali metals has grown steadily and strongly over the past several years.^[1]^ New unconventional applications of the heavier homologs of lithium are increasingly being published, challenging the indispensability of organolithium chemistry.^[2]^ Especially, hydroaminations with polar organometallic catalysts of group 1 and 2 are attracting much attention.^[3]^ Nevertheless, besides the lower stabilities and poorer solubility of the corresponding organyls,^[4]^ the accessibility of the reagents also plays a crucial role for the use of the heavy alkali metals in synthetic chemistry. In most common cases, the so‐called Lochmann‐Schlosser or LICKOR base is often used to introduce heavy alkali metals into substrates.^[5]^ However, this can lead to mixed alkoxide aggregates that change the stoichiometry of the metalation reagent and require an excess of the alkali metal alkoxide used, which in turn can lead to the formation of by‐products.[^5d^, ^6^] Therefore, it is all the more desirable to synthesize defined monometallic reagents that exhibit a compromise between stability and reactivity so that they can be isolated on the one hand and used for reactions on the other.^[7]^ So far, only a few crystalline examples of well‐defined heavy alkali metal compounds are known. Most of these are synthesized by using a Lochmann‐Schlosser base, although it does not directly pave the way to defined reagents.^[8]^ Hence, amine‐based chelating ligands are often used for separation of impurities by crystallization and to obtain aggregates that are as small as possible and at the same time very stable and defined.[^9^, ^10^, ^11d^, ^12^] Examples with exclusively etheric ligands are even rarer, as these are often much more unstable in solution and thus more difficult to isolate due to metalation of the etheric solvent and subsequent cleavage. Isolated crystals often show the detachment of THF with accompanying loss of crystallinity.[^5d^, ^9^, ^10a^, ^11^, ^13^, ^14^] Considering these reagents, one class of compounds attracts attention, which combines the compromise of reactivity and stability: The alkali metal benzyl compounds. However, except for THF‐solvated benzyl potassium,[^5d^, ^13^] there are only defined heavy alkali metal benzyl compounds in combination with polyamine ligands known.[^12b^, ^12c^] As shown in previous work, ether‐solvated alkali metal deprotonation compounds can help to understand deprotonation mechanisms exchanging the coordinating ether with a substrate.^[5d]^ Therefore, it is important to gain insight into the structure of deprotonation reagents.

Within this paper, selective synthesis of THF‐solvated metal benzyl compounds of the alkali metals sodium, rubidium and caesium is demonstrated for the first time (Scheme 1). In addition, the molecular structures of these complexes in the solid state and their application in aminometalation reactions is presented.

**Scheme 1:**
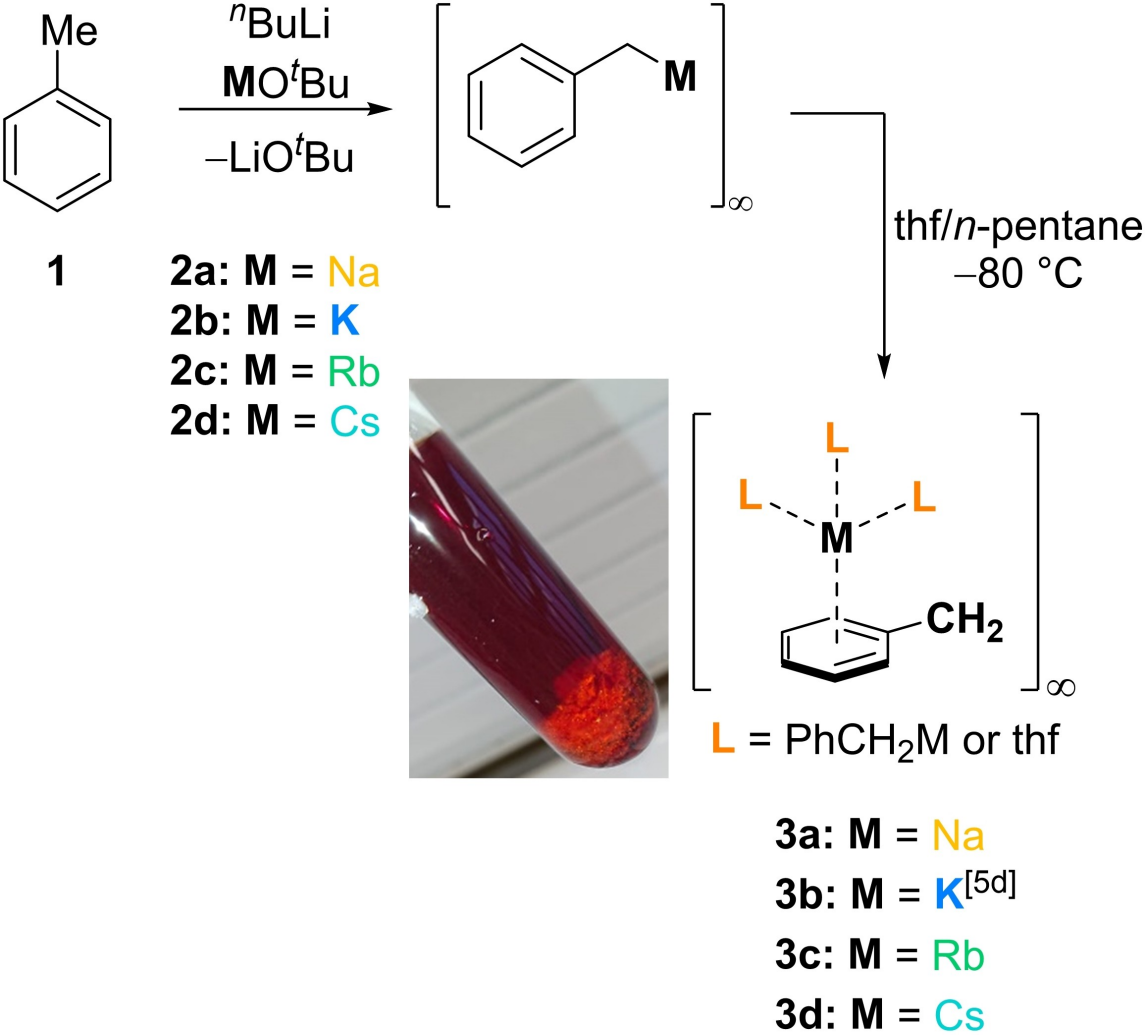
Synthesis of THF‐solvated heavy alkali metal benzyl compounds presented in this and previous works.^[5d]^ Inset picture shows the thf‐adduct **3 b** after dissolution of the solid in THF and layering with *n*‐pentane.

The deprotonation of toluene (**1**) with a bimetallic mixture of an alkali metal *tert*‐butoxide (M=Na, Rb, Cs) and *n*‐butyllithium was carried out to obtain the corresponding benzyl metal compounds **2 a**, **2 c** and **2 d** as solids or as solutions in THF. Subsequent dissolution of the solids in fresh THF, layering the THF‐solutions with *n*‐pentane and storage at −80 °C for several days resulted in the formation of the THF‐solvated compounds **3 a**, **3 c** and **3 d**. The benzyl‐sodium THF‐solvate **3 a** crystallizes in a mixture of THF and *n*‐pentane in the orthorhombic crystal system, space group *P*2_1_2_1_2_1_ (Figure 1). The asymmetric unit contains the fragment [(PhCH_2_Na)(thf)_2_]. The intermediate forms a 1D coordination polymer with a zig‐zag structure via η^2^‐binding modes of the sodium center to C1 and C2 carbon center of the benzyl anions. Each of the metal centers is coordinated by two benzyl moieties and two THF molecules.

**Figure 1:**
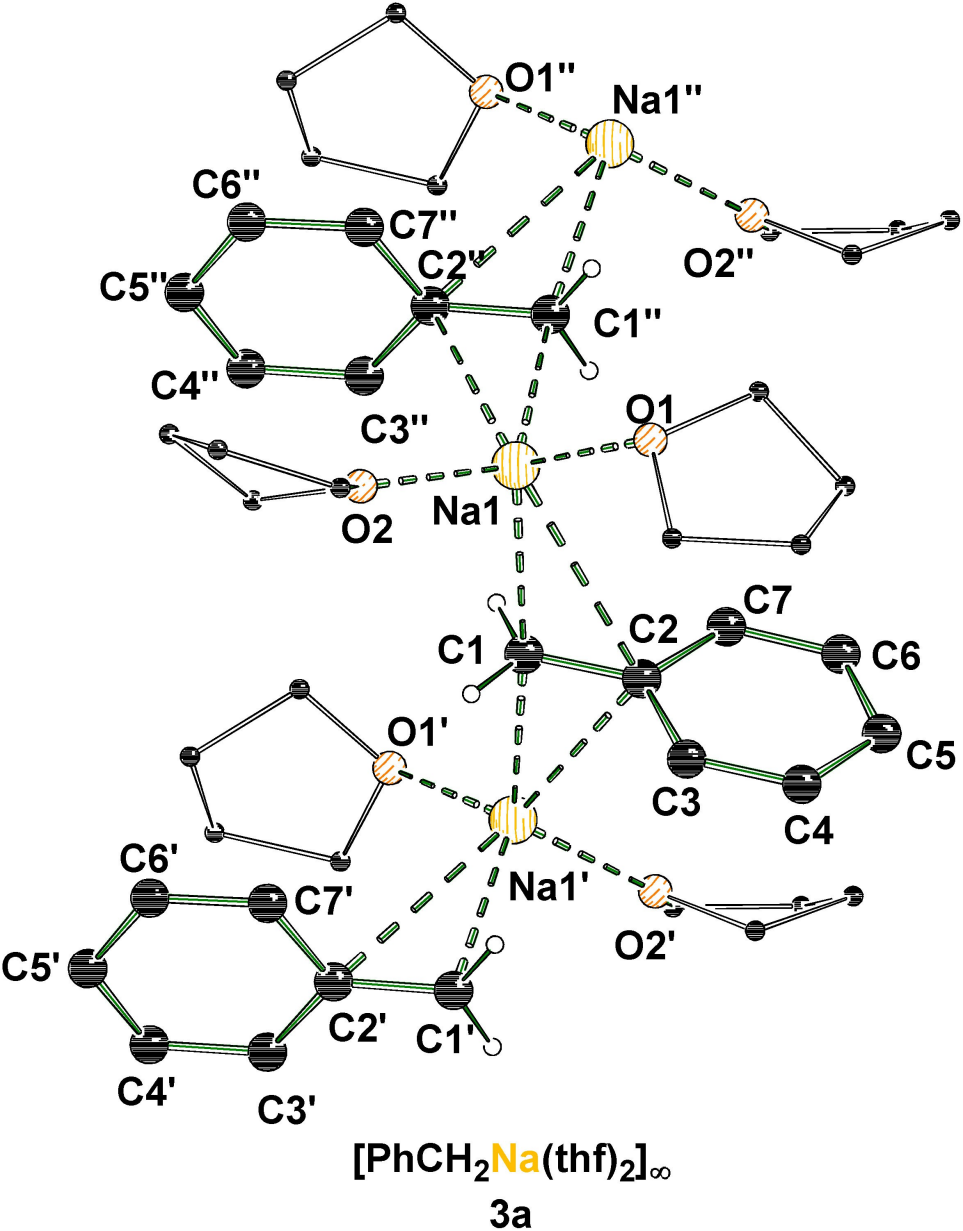
Polymeric structure of [(PhCH_2_Na)(thf)_2_]_∞_ (**3 a**; some hydrogen atoms are omitted for clarity). Symmetry‐equivalent positions are included to visualize the coordination environment of the sodium centers (#1’:1/2+x, 1/2−y, 1−z; #2’’: −1/2+x, 1/2−y, 1−z). For further information see Supporting Information.

A similar coordination environment can be found in the reactive intermediate **3 c**. The benzyl‐rubidium THF‐solvate **3 c** crystallizes in a mixture of THF and *n*‐pentane in the orthorhombic crystal system, space group *Pna*2_1_ (Figure 2). Similar to **3 a** the asymmetric unit of **3 c** contains one metal center, coordinated by one benzyl anion and two THF molecules, forming the fragment [(PhCH_2_Rb)(thf)_2_]. Further, the intermediate forms a 1D coordination polymer that is built up by multi η‐interactions between metal and anion. For each rubidium cation two contacts to benzyl anions as well as two contacts to THF molecules can be observed.

**Figure 2:**
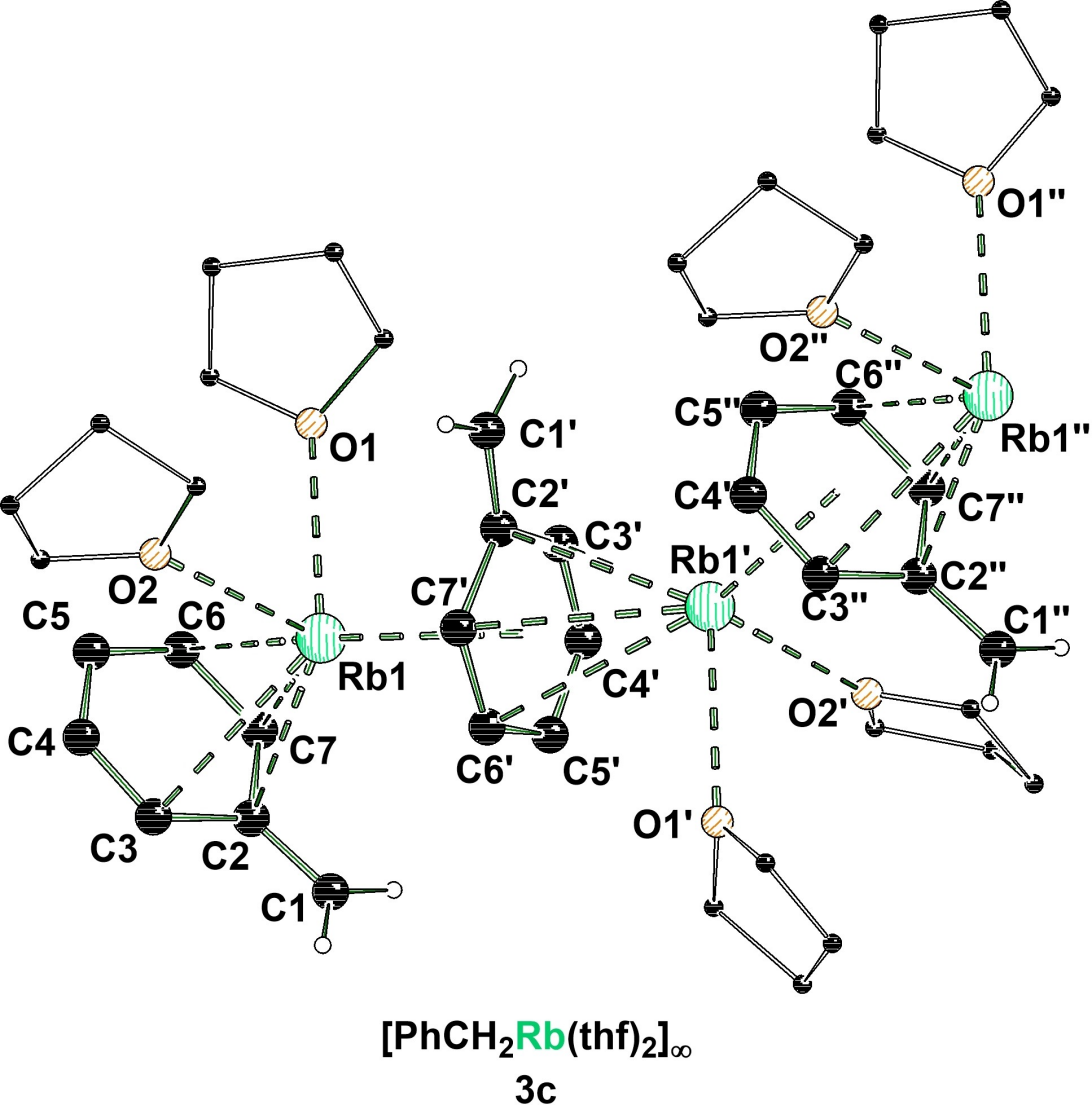
Polymeric structure of [(PhCH_2_Rb)(thf)_2_]_∞_ (**3 c**, some hydrogen atoms are omitted for clarity). Symmetry‐equivalent positions are included to visualize the coordination environment of the rubidium centers (#1’: −x, −y, 1/2+z; #2’’: x, y, 1+z). For clarity, disorders of the THF molecules are omitted. For further information see Supporting Information.

An important distinction between the two polymeric structures of **3 a** and **3 c** is the coordination mode of the anion to the metal. While in the sodium compound **3 a** the contacts to the metal occur predominantly via the *alpha*‐ and *ipso*‐carbon centers, in the rubidium compound **3 c** the contact is formed exclusively with carbon centers of the phenyl ring.

In a strong contrast to these two complexes is the benzylcaesium THF‐solvate **3 d**, which crystallizes in a mixture of THF and *n*‐pentane in the monoclinic crystal system, space group *C*2/*c* (Figure 3). The asymmetric unit contains the anion and half a THF molecule coordinating the caesium cation. On closer examination of the compounds **3 c** and **3 d** a major difference appears moving from rubidium to caesium. The structure in the solid‐state grows from a 1D to a 2D coordination polymer with an accompanying loss of one THF molecule. As a result, each metal center features two η^1^‐contacts, a η^3^‐contact and a η^6^‐contact to a total of four benzyl anions as well as one coordination of a THF molecule.

**Figure 3:**
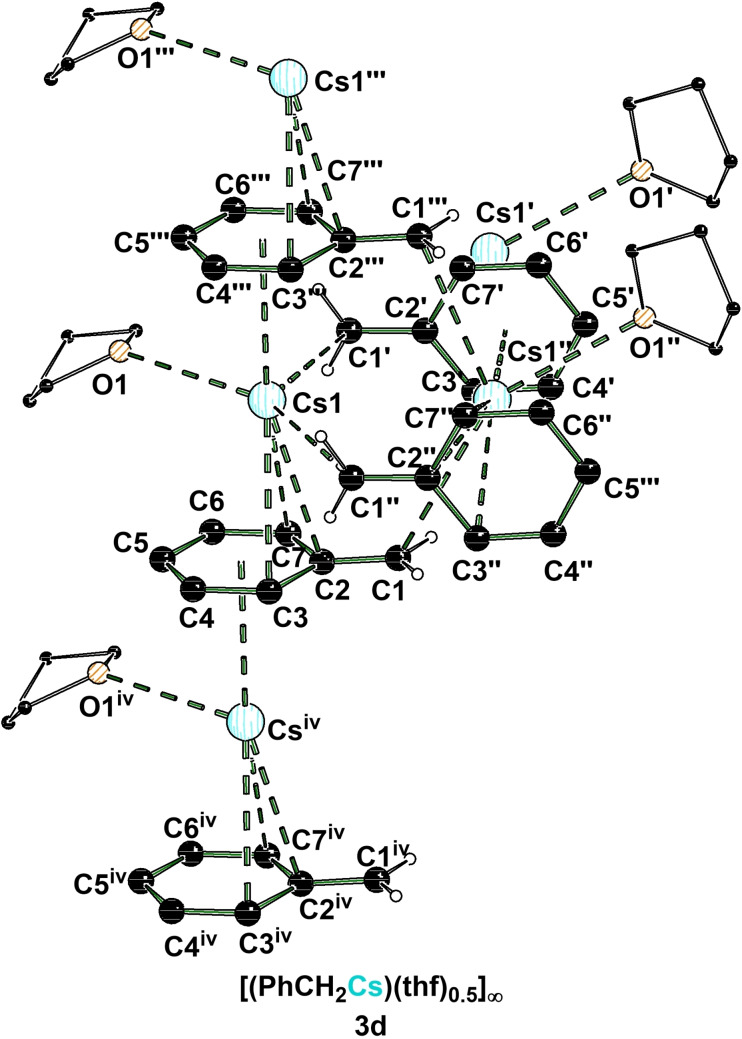
Polymeric structure of [(PhCH_2_Cs)(thf)_0.5_]_∞_ (**3 d**; some hydrogen atoms are omitted for clarity). Symmetry‐equivalent positions are included to visualize the coordination environment of the caesium centers (#1’: 1/2−x, 1/2+y, 1/2−z; #2’’: 1−x, y, 1/2−z; #3’’’: 1/2+x, 1/2+y, z; #4^iv^: −1/2+x, −1/2+y, z). For further information see Supporting Information.

As also described in previous work, the stronger charge delocalization on the benzyl anion with descending group 1 can be observed in the solid state, as well as in solution.^[12]^ For this purpose, ^1^H NMR and ^13^C NMR spectra of **3 a**, **3 c** and **3 d** in THF‐d_8_ were recorded (Table 1). Only one set of signals was observed for all species, indicating a symmetrical environment for the benzyl anions in the present aggregates in solution. However, significant distinctions are evident depending on the metal. Moving from Na to Cs, the ^1^H NMR spectra reveal a proton‐shift of the metalated carbon center to the low field, whereas the protons of the aryl ring shift to the high field. In the ^13^C NMR spectra, this trend is only revealed by the shifts of the *alpha*‐ as well as the *para*‐carbon centers. In addition to the NMR shifts, the bond lengths in the solid state also suggest a charge delocalization in the benzyl anion depending on the metal. Consequently, the C_*α*_−C_*ipso*_ and C_*ortho*_−C_*meta*_ bonds are shortened with increasing radius of the cation, whereas the C_*ipso*_−C_*ortho*_ and C_*meta*_−C_*para*_ bonds are elongated (for more details see Supporting Information).

**Table 1:** NMR resonances in THF‐d_8_ of the complexes **3 a**, **3 b**, **3 c** and **3 d** compared with benzyllithium (all values in ppm).

**Compound**	**Benzyllithium^[15]^**	**3 a**	**3 b^[16]^**	**3 c** ^[a]^	**3 d** ^[a]^
nuclei	^1^H; ^13^C	^1^H; ^13^C	^1^H; ^13^C	^1^H; ^13^C	^1^H; ^13^C^]^
*alpha*	1.61: 37.1	1.93; 41.1	2.24; 52.7	2.25; 54.6	2.29; 56.3
*ipso*	–; 161.2	–; 157.7	–; 152.9	–; 153.2	–; 154.1
*ortho*	6.12; 116.9	5.85; 113.4	5.60; 111.1	5.47; 111.4	5.35; 112.7
*meta*	6.36; 128.4	6.14; 126.3	6.12; 130.7	6.02; 130.9	5.96; 131.0
*para*	5.46; 104.7	5.02; 100.4	4.80; 95.9	4.70; 95.2	4.68: 94.6

[a] ^1^H NMR shifts are in good agreement with the results of Harder *et* *al*.^[8d]^

To gain a deeper insight into the electronic situation of the THF‐solvated alkali metal benzyl species, monomeric as well as dimeric aggregates of the benzyl compounds were modeled and optimized on the B3LYP‐D3/def2tzvpp level of theory. Furthermore, NBO charges on the B3LYP/def2tzvpp level of the different benzyl units in the dimer were determined, allowing the monitoring of asymmetric charge distribution and associated bond length changes in the aryl ring, which are in good agreement with the experimentally observed bond length in the solid state (for more details see Supporting Information). As with the NMR shifts, the largest discrepancy is observed in the change from sodium to potassium, which is why only the results for these two metals are presented here (Figure 4). The results of the heavier homologs are similar to those of potassium (see Supporting Information). It is striking that asymmetric interactions with the cationic metal centers result in an asymmetric charge distribution in the benzyl anion. This asymmetry can also be observed in the solid‐state structures of the benzyl rubidium solvate **3 c** or the benzyl potassium THF solvate of our previous work **3 b** ^[5d]^ and is best reproduced by the modeled dimer with a sandwiched benzyl anion. The opposite leads to a symmetrical charge distribution, as it is the case in the benzyl sodium solvate **3 a**, which in turn is better illustrated by the modeled monomer or the isolated benzyl anion in the dimer.

**Figure 4:**
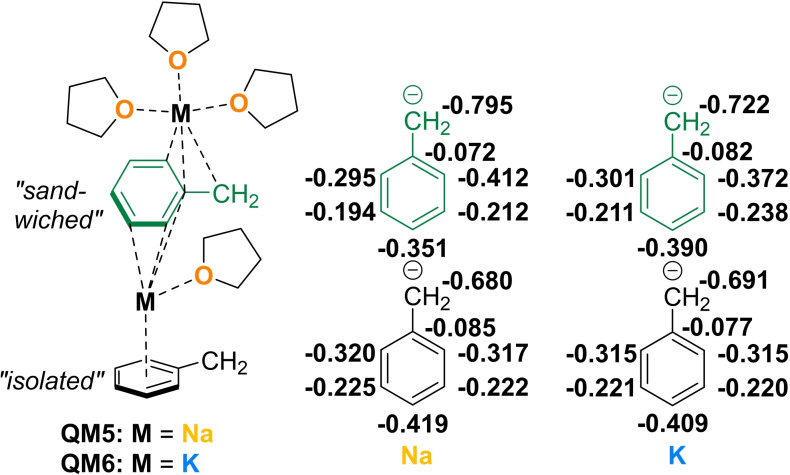
Comparison of the NBO charges in the “sandwiched” and “isolated” benzyl anion depending on the metal (M= Na, K). Level of theory B3LYP/def2tzvpp. For information about the monomers and the rubidium/caesium dimers see Supporting Information.

With completion of the THF‐solvate benzyl compounds of the lithium congeners and insights into their structure in the solid‐state, in solution as well as in their electronic structure, we have set out to explore the capability to mediate the alkali metal to substrates. Recently, our group showed the aminometalation and subsequent functionalization of diphenylethene with piperidine mediated by mixed potassium/lithium amides.^[3d]^ In this study the aminometalation by monometallic amides, prepared by the deprotonation with the well‐defined benzyl metal solvates, was investigated. First it was important to assess the stability of the deprotonated aminometalation product with the heavier congeners of potassium. As the most stable substrate in the previous study *N*,*N*‐dimethyl‐2,2‐diphenylethan‐1‐amine (**4**) was chosen for the deprotonation reaction. Deprotonation of **4** was achieved by using benzyl caesium solvate **3 d**, which is reflected by an immediate change of the color from red to dark red.^[17]^ Storage of the solution at −80 °C leads to the formation of orange‐red blocks of intermediate **5** (Scheme 2).

**Scheme 2:**
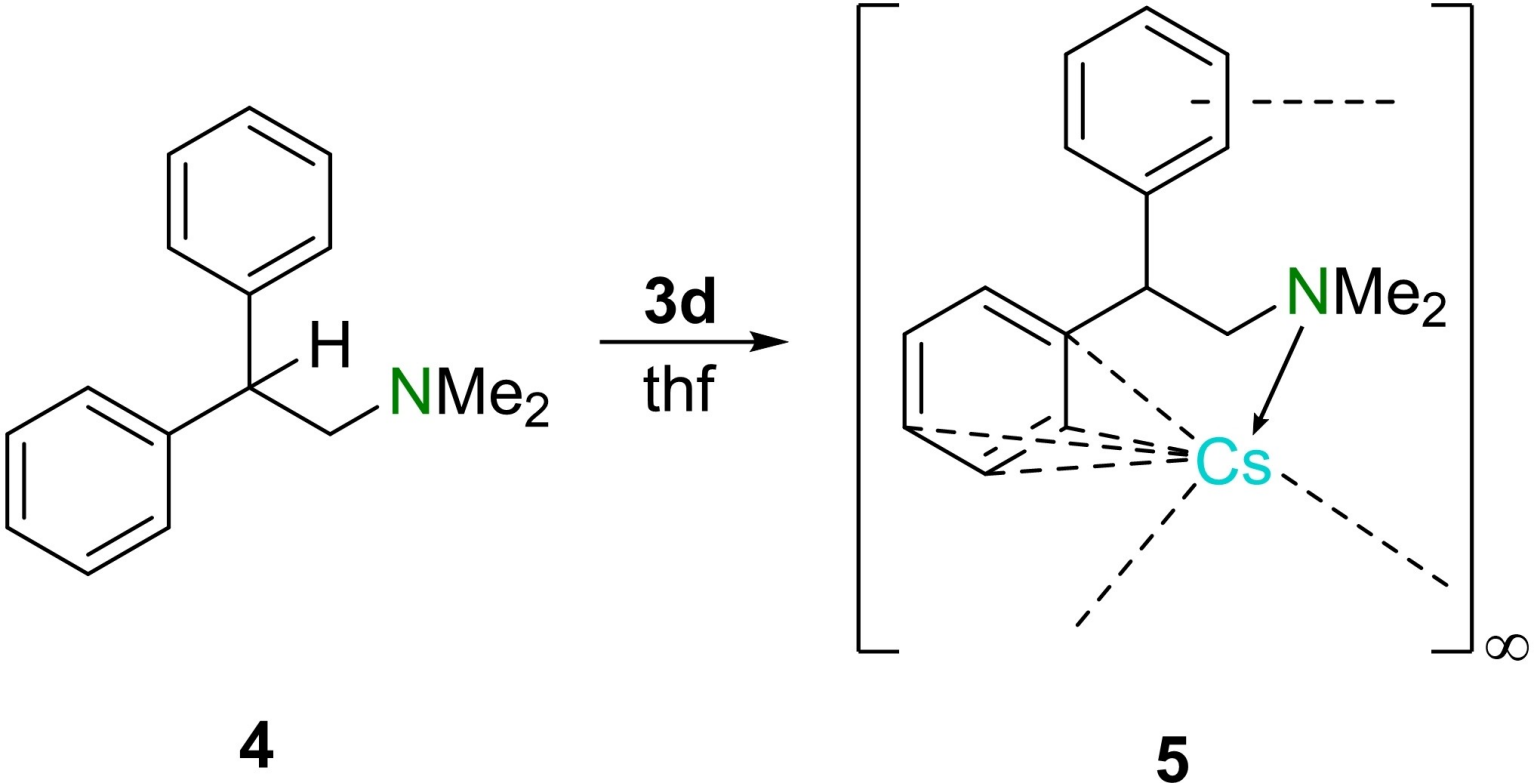
Deprotonation of *N*,*N*‐dimethyl‐2‐phenylethan‐1‐amine (**4**) with THF‐solvated benzylcaesium **3 d**.

The intermediate crystallizes in THF in the rhombohedral space group *R* $\bar{3}$ . In the solid state it forms a polymeric 3D structure, where the metal center is coordinated by the nitrogen center of the aminomethyl‐handle and in total by three anions via two η^4^‐ and one η^3^‐contact (Figure 5). The negative charge delocalization across the phenyl ring C2‐C7 in plane to the carbanionic center is indicated by significant deformation in contrast to the phenyl ring C8‐C13. In addition, toluene co‐crystallizes to the metalated species **5** as a result of the deprotonation and noticeably no coordinating THF can be found in the solid‐state structure. This underlines the prioritization of the heavier alkali metals for anions over polar ligands such as THF.^[12b]^ The solution phase NMR spectra of this compound reveal a single set of signals, indicating a symmetric diphenylmethanide in the present aggregate. The characteristic shifts in the ^1^H‐ and ^13^C NMR spectra of the aromatic system are also in good agreement with comparable metalated aminometalation products of our previous study.^[3d]^ Hence, it should be possible to introduce the metal by an aminometalation of the alkali metal amide and the corresponding alkene. To confirm this hypothesis, the reaction of piperidine (**6**) with the alkali metal benzyl compounds **3 b**,^[5d]^ **3 c** or **3 d** and 1,1‐diphenylethene was carried out. Afterwards the reaction was trapped with *n*‐butyl bromide, which was followed by GC/EI‐MS and NMR spectroscopy, to give the desired β‐functionalized product **7** (Scheme 3).

**Figure 5:**
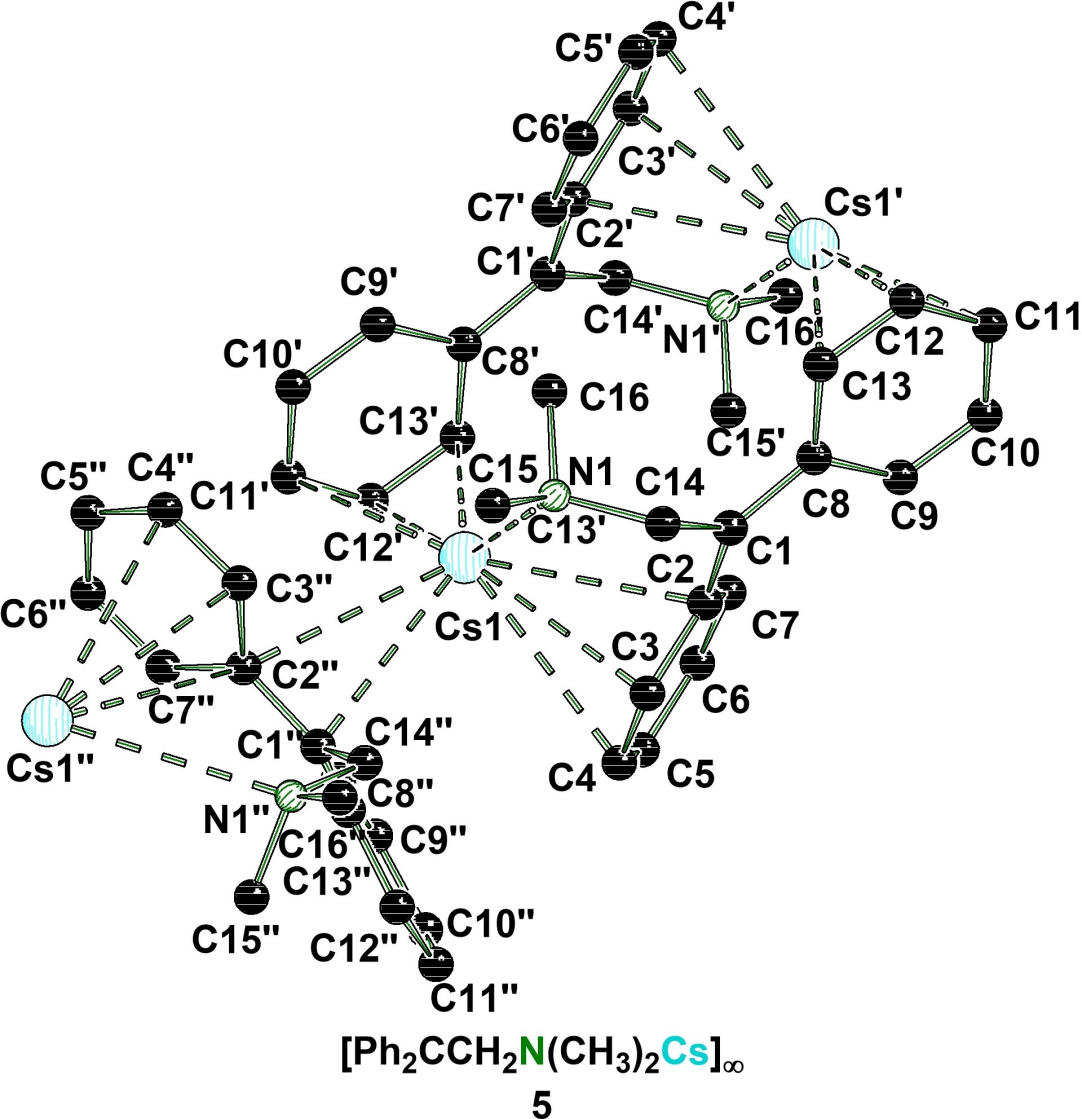
Solid state structure of **5** by deprotonation of **4** with **3 d**. Hydrogen atoms and disorder are omitted for clarity. Co‐crystallized toluene has been squeezed. (#1’: 1−x, 1−y, 1−z; #2’’: −1/3+y, 1/3−x, 4/3−z). For further information see Supporting Information.

**Scheme 3:**
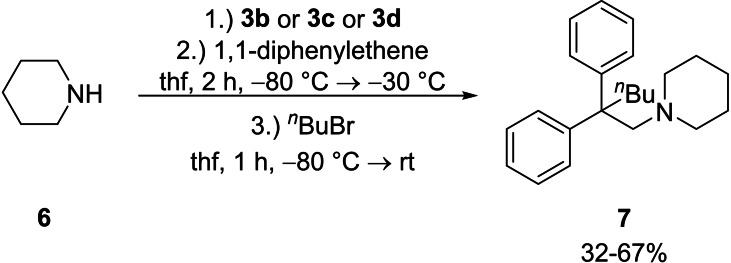
“One pot” synthesis of **7** by the *in* *situ* formation of the metal piperidides, the addition to the alkene and the trapping reaction of the metalated species with *n*‐butyl bromide.

The reaction reveals that the synthesis of functionalized aminometalation products is also possible with monometallic heavy alkali metal amides prepared, by the deprotonation with well‐defined benzyl metal reagents in THF. The use of a second metal or alkoxides does not come into play here. The isolation of the reactive species **5** and the aminometalation reaction followed by functionalization provide new starting points to elucidate the mechanism of this reaction. Therefore, the THF‐solvated alkali metal benzyl compounds may become important mediators to synthesize catalysts for hydroamination reactions.

In summary, the selective synthesis of previously unknown THF‐solvated alkali metal benzyl compounds (Na, Rb, Cs) and thus the completion of this attractive substance class for group 1 is demonstrated. Insights into the molecular structure in the solid‐state as well as in solution can be obtained by single‐crystal X‐ray structure analysis and NMR spectroscopy. Quantum chemical calculations contribute to the understanding of the asymmetric coordination modes of specific metals, obtained in the solid‐state, affecting the charge distribution in the benzyl anion. In particular, the potential to prepare metal amides by alkali metal mediated deprotonation without polyamine ligands, second metals, or alkoxides, and the easier handling compared to alkali metal alkyl compounds highlight these compounds. However, the mediation can be applied not only to N−H, but also to C−H acidic compounds to prepare new organometallic species.

## Experimental Section

For experimental details, see the Supporting Information.

**X‐ray crystallography**: Deposition Number(s) 2051649 (for **3 a**), 2051670 (for **3 c**), 2051720 (for **3 d**) and 2051743 (for **5**) contain(s) the supplementary crystallographic data for this paper. These data are provided free of charge by the joint Cambridge Crystallographic Data Centre and Fachinformationszentrum Karlsruhe Access Structures service.

## Conflict of interest

The authors declare no conflict of interest.

## Supporting information

As a service to our authors and readers, this journal provides supporting information supplied by the authors. Such materials are peer reviewed and may be re‐organized for online delivery, but are not copy‐edited or typeset. Technical support issues arising from supporting information (other than missing files) should be addressed to the authors.

Supporting Information
