# Supplementary material for: THF‐solvated Heavy Alkali Metal Benzyl Compounds (Na, Rb, Cs): Defined Deprotonation Reagents for Alkali Metal Mediation Chemistry
Source: Chemistry. 2021 Oct 25;27(71):17780–4. doi: 10.1002/chem.202103430 (PMC9298144; doi:10.1002/chem.202103430)
Supplement: Supplementary file 1 — Supporting Information [file CHEM-27-17780-s001.pdf]

Chemistry–A European Journal

Supporting Information

THF-solvated Heavy Alkali Metal Benzyl Compounds (Na, Rb, Cs): Defined Deprotonation Reagents for Alkali Metal Mediation Chemistry

Lukas Brieger, Christian Unkelbach, and Carsten Strohmann*

Table of Content

1. General Remarks **3**

2. Experimental Procedures **4**

2.1 General experimental procedure for the handling of 3a-d 4
2.2 One pot synthesis of 1-(2,2-diphenylhexyl)piperidine (7) 4

3. Single crystal X-ray diffractional analysis **5**

3.1 Synthesis of the benzylsodium thf-solvate 3a 6
3.2 Synthesis of the benzylrubidium thf-solvate 3c 8
3.3 Synthesis of the benzylcaesium thf-solvate 3d 11
3.4 Synthesis of metalated compound 5 13
3.5 Re-recorded NMR spectra of compound 3b 19
3.6 Decomposition of the thf-solvated benzyl compounds in thf at room temperature 20

4. Quantum Chemical Calculations **21**

4.1 Calculated Monomers of Alkali Metal Benzyl Compounds coordinated by thf 21
4.2 Calculated Dimers of Alkali Metal Benzyl Compounds coordinated by thf 28
4.3 Data of the calculated monomeric and dimeric benzyl compounds 36
4.3.1 Summary 39
4.4 Isodesmic reaction towards the deprotonation of *N*,*N*-Dimethyl-2,2-diphenylethan-1-amine (XX) 41

5. References **44**

1. General Remarks

All reactions with oxygen- and moisture-sensitive compounds were performed under an atmosphere of argon in dried solvents, which were distilled prior to use. Deuterated thf (thf-d_8) was dried over sodium. All other solvents and commercially available reagents, including the NMR solvents, were used without further purification.

The NMR spectra were measured on a *Bruker Avance DRX-400* and on a *Bruker Avance DRX-500* NMR spectrometer. All NMR spectra were recorded at room temperature (ca. 25 °C). Chemical shifts (δ in ppm) are referred to tetramethylsilane (TMS), with the deuterium signal of the solvent serving as internal lock and the residual solvent signal as additional reference [^{1}H-NMR: $\delta(C_6D_5H)$ = 7.16, $\delta(C_4D_7HO)$ = 1.72; 3.58, $\delta(CHCl_3)$ = 7.26; ^{13}C-NMR: $\delta(C_6D_6)$ = 128.4, $\delta(C_4D_7HO)$ = 25.3; 67.2, $\delta(CDCl_3)$ = 77.0]. The ^{29}Si-NMR experiments are referred to TMS as external standard and measured via the INEPT puls sequence. For the assignment of the multiplicities the following abbreviations were used: s = singlet, d = doublet, t = triplet, m = multiplet, br = broad signal. Aromatic carbon and hydrogen atoms were assigned as follows: i = ipso, o = ortho, m = meta, p = para.

GC/EI-MS analyses were obtained using an Agilent 7890B GC system (column: Agilent HP-5MS, 30 m, 0.25 mm, 0.25 µm) with an Agilent 5977A Mass Selective Detector.

Suitable crystals of compound **3a**, **3c**, **3d**, and **5** were covered with an inert oil (perfluoroalkylether) at –80 °C using the *X-TEMP 2*[1] device in combination with a *SMZ1279* stereomicroscope from *Nikon Metrology GmbH* and mounted on a *MicroMount* from *MiTeGen*. Crystal structure determination was accomplished on a *Bruker D8 Venture* four-circle diffractometer using a *PHOTON II CPAD* detector by *Bruker AXS GmbH*. X-ray radiation was generated by microfocus source *IµS* Mo (λ = 0.71073 Å) by *Incoatec GmbH* with HELIOS mirror optics and a single-hole collimator by *Bruker AXS GmbH*. For the data collection, the programs APEX 3 Suite (v.2018.7-2) with the integrated programs SAINT (integration) and SADABS (adsorption correction) by *Bruker AXS GmbH* were used. The processing and finalization of the crystal structure was done with the program Olex2.[2] The crystal structure was solved with the ShelXT[3] structure solution program using Intrinsic Phasing and refined with the ShelXL refinement package using Least Squares minimization. The non-hydrogen atoms were refined anisotropically. U_{eq} is defined as one third of the trace of the orthogonalized tensor U_{ij}. For the hydrogen atoms the standard values of the SHELXL[4] program were used with $U_{iso}(H) = -1.2\ U_{eq}(C)$ for CH_2 and CH and with $U_{iso}(H) = -1.5\ U_{eq}(C)$ for CH_3.

2. Experimental Procedures

2.1 General experimental procedure for the handling of 3a-d

At –80 °C the crystalline compounds of **3a**–**3d** were separated from the mother liquor and washed at constant temperature with cold *n*-pentane until the solvent remained colorless. Subsequently, the crystals were carefully dried in vacuo for a short time and taken up in cold, fresh thf, in which they initially remained crystalline. In the purification process the temperature should not exceed –50 °C After adding the first substrate and warming up to the desired temperature, the crystals dissolve. This procedure avoids by-products that could arise from thf-cleavage reactions.

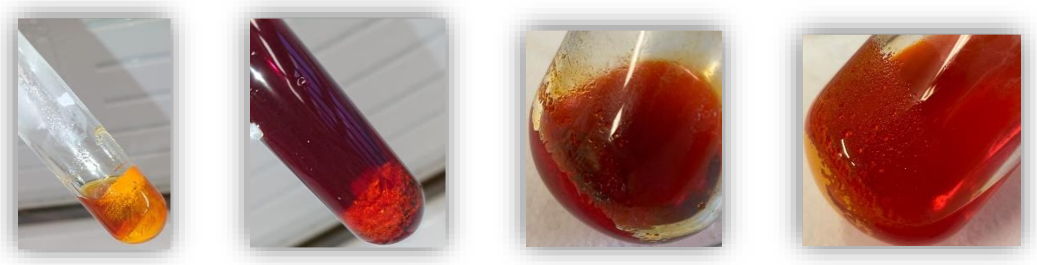

Figure S1 Images of the crystalline compounds **3a–3d**.

2.2 One pot synthesis of 1-(2,2-diphenylhexyl)piperidine (7)

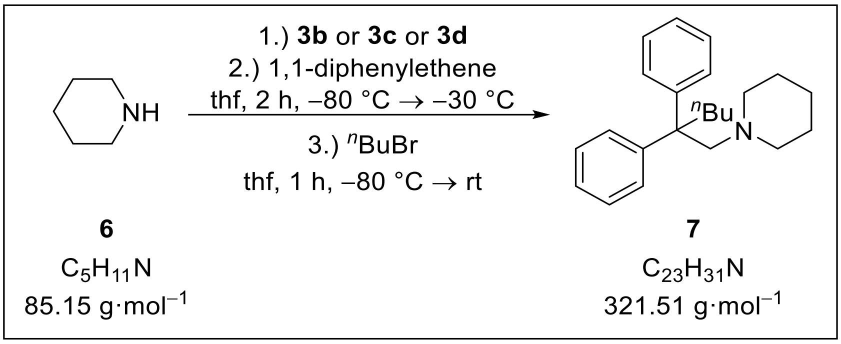

To a stirred suspension of freshly prepared **3b**[5]**-3d** (K: 272 mg; Rb: 318 mg; Cs: 296 mg, 1.00 mmol, 1.0 eq) in thf, piperidine (0.10 ml, 1.00 mmol, 1.0 eq.) was added at –80 °C. The solution was stirred for one hour, allowed to warm up to –30 °C and during this time it changed its color from dark red to red-brown. Subsequently, the solution was cooled to –80 °C and 1,1-diphenylethene (0.18 ml, 1.00 mmol, 1.0 eq.) was added. The reaction solution turned red after a couple of minutes, was stirred for one hour and was allowed to warm up to –30 °C. Afterwards, *n*-butylbromide (0.21 ml, 2.00 mmol, 2.0 eq.) was added at –80 °C and the red solution discolored immediately. The reaction mixture was stirred one more hour and was allowed to warm up to room temperature. After adding hydrochloric acid (5 ml, 2.0 M) and distilled water (5 ml), the aqueous phase was separated and treated with KOH (2.0 M) until the pH was at 13. The organic layer formed was separated and the aqueous phase was extracted with diethylether (3 x 10 ml). The combined organic layers were dried with $MgSO_4$ and the solvent was removed. The functionalized aminometalation product **7** was obtained as colorless residue with a yield of K:215 mg, Rb: 143 mg and Cs: 103 mg (k: 0,67 mmol, 67%; Rb: 0.45 mmol, 45%; Cs: 0.32 mmol, 32%). The analytical data for this

compound are in good agreement with our already published results.[6] The declining yields correlate with the poorer solubility moving from thf-solvated benzyl potassium to benzyl caesium.

^{1}H-NMR (400.25 MHz, $CDCl_3$, 298 K): δ = 0.83 (t, $^3J_{H,H}$ = 7.3 Hz, 3H; $CH_2CH_2CH_2CH_3$), 0.88 – 0.92 (m. 2H; $CH_2CH_2CH_2CH_3$), 1.24 – 1.33 (m, 8H; $NCH_2CH_2CH_2$ und $CH_2CH_2CH_2CH_3$), 1.99 (t, $^3J_{H,H}$ = 5.5 Hz, 4H; $NCH_2CH_2CH_2$), 2.20 – 2.25 (m, 2H; $CH_2CH_2CH_2CH_3$), 2.93 (s, 2H; NCH_2C), 7.12 – 7.22 (m, 10H; H_{ar}) ppm.

{^{1}H}^{13}C-NMR (100.64 MHz, $CDCl_3$, 298 K): δ = 14.2 (1C; $CH_2CH_2CH_2CH_3$), 23.6 (1C; $CH_2CH_2CH_2CH_3$), 24.3 (1C; NCH2CH2*C*H2), 26.7 (2C; NCH2*C*H2CH2), 26.9 (1C; $CH_2CH_2CH_2CH_3$), 36.1 (1C; $CH_2CH_2CH_2CH_3$), 50.8 (1C; NCH2*C*Ph2), 56.9 (2C; NCH2CH2CH*2*), 65.3 (1C; NCH2C), 125.5 (2C; *Cpara*), 127.6 (4C; *Cortho*), 128.6 (4C; *Cmeta*), 148.7 (2C; *Cipso*) ppm.

GC/EI-MS Methode E, (70 eV, t_R = 17.26 min,): *m/z* (%) = 320 (1) [(M-H)$^+$], 264 (1) [$(C_{19}H_{22}N)^+$], 222 (1) [$(C_{17}H_{18})^+$], 165 (8) [$(C_{13}H_9)^+$], 98 (100) [$(C_6H_{12}N)^+$], 91 (6) [$(C_7H_7)^+$].

3. Single crystal X-ray diffractional analysis

Crystallographic data for the structures of **3a**, **3c**, **3d**, and **5** have been deposited with the Cambridge Crystallographic Data Centre as supplementary publication numbers (Table S1). Copy of these data can be obtained, free of charge, on application to CCDC, 12 Union Road, Cambridge CB2 IEZ, UK, fax: 144-(0)1223-336033 or e-mail: deposit@ccdc.cam.ac.uk.

Table S1 CCDC deposition numbers for the crystallized compounds.

Compound	CCDC deposition number
**3a**	2051649
**3c; modification**	2051670; 2052133
**3d**	2051720
**5**	2051743

3.1 Synthesis of the benzylsodium thf-solvate 3a

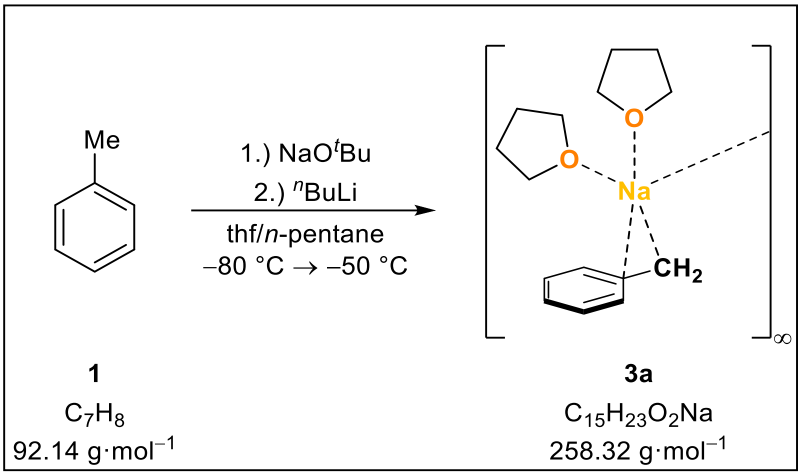

To a stirred solution of toluene (92 mg, 1.0 mmol, 1.0 eq.) and sodium *tert*-butoxide (0.5 mL, 1.0 mmol, 1.0 eq, 2.0 M in thf) at –80 °C *n*-butyllithium (0.44 ml, 1.1 mmol, 1.1 eq. 2.5 M in hexane) was added. A yellow solution was obtained, which was allowed to warm up to –50 °C. It was layered with *n*-pentane (1.0 ml) and stored at –80 °C. After six days yellow needles of compound **3a** formed, suitable for single crystal X-ray analysis. The mother liquor was removed and the crystals were washed with cold *n*-pentane (3 x 1 ml) until the solvent remained colorless. Afterwards the crystals were dried in the vacuum, but due to the weak thf-metal bond decomposition of the crystals at room temperature occurred. At low temperatures a yield of 198 mg (77%, 0.77 mmol) could be estimated. Some solvent-based impurities can be observed in the NMR spectra as well. However, the most characteristic signals could be characterized, like the highly shifted benzylic and aromatic signals in the ^{1}H- and ^{13}C-NMR spectra.

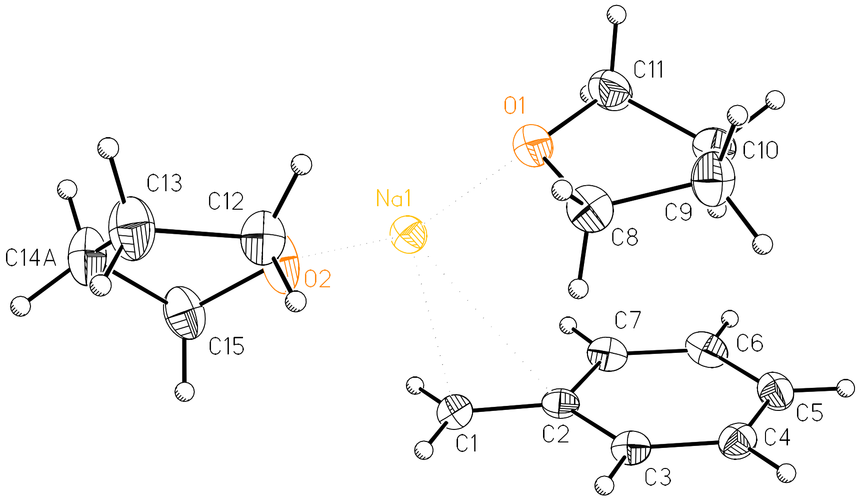

3a

Figure S2 Ortep plot[7] of the asymmetric unit of **3a** in the crystal, with displacement ellipsoids drawn at the 50% probability level. Numbering scheme of hydrogen atoms and disorders are omitted for clarity. Selected bond lengths [Å] and angles [°]: Na1–O1 2.195(1), Na1–O2 2.271(1), Na1–C1 2.543(2), Rb1–C2 3.113(2), C1–C2 1.411(2), C2–C3 1.436(2), C2–C7 1.433(2), C3–C4 1.384(2), C4–C5 1.397(2), C5–C6 1.396(2), C6–C7 1.380(2); C1–C2–Na1 53.6(1), C1–C2–C3 123.2(1), C1–C2–C7 122.7(1), C3–C2–C7 114.1(1), C4–C3–C2 122.4(1), C3–C4–C5 121.8(1), C4–C5–C6 117.3(1), C5–C6–C7 121.9(1), C6–C7–C2 122.5(1), O1–Na1–O2 98.2(1).

^{1}H-NMR (400.1 MHz, thf-d$_8$, 298 K): δ = 1.76-1.79 [m, 16H; β-CH_2 (thf)], 1.93 [s, 2H; C(CH_2)], 3.60-3.64 [m, 16H; α-CH_2 (thf)], 5.02-5.06 [m, 1H; H$_{para}$], 5.85-5-89 [m, 2H; H_{ortho}], 6.14-6.18 [m, 2H; H_{meta}] ppm.

{^{1}H}^{13}C-NMR (100.64 MHz, thf-d$_8$, 298 K): δ = 26.6 [8C; β-CH$_2$ (thf)] 41.1 (1C, CNa*C*H$_2$), 68.4 [8C; α-CH$_2$ (thf)], 100.4 (1C; C_{para}), 113.4 (2C; C_{ortho}), 126.3 (2C; C_{meta}), 157.7 (1C; C_{ipso}) ppm.

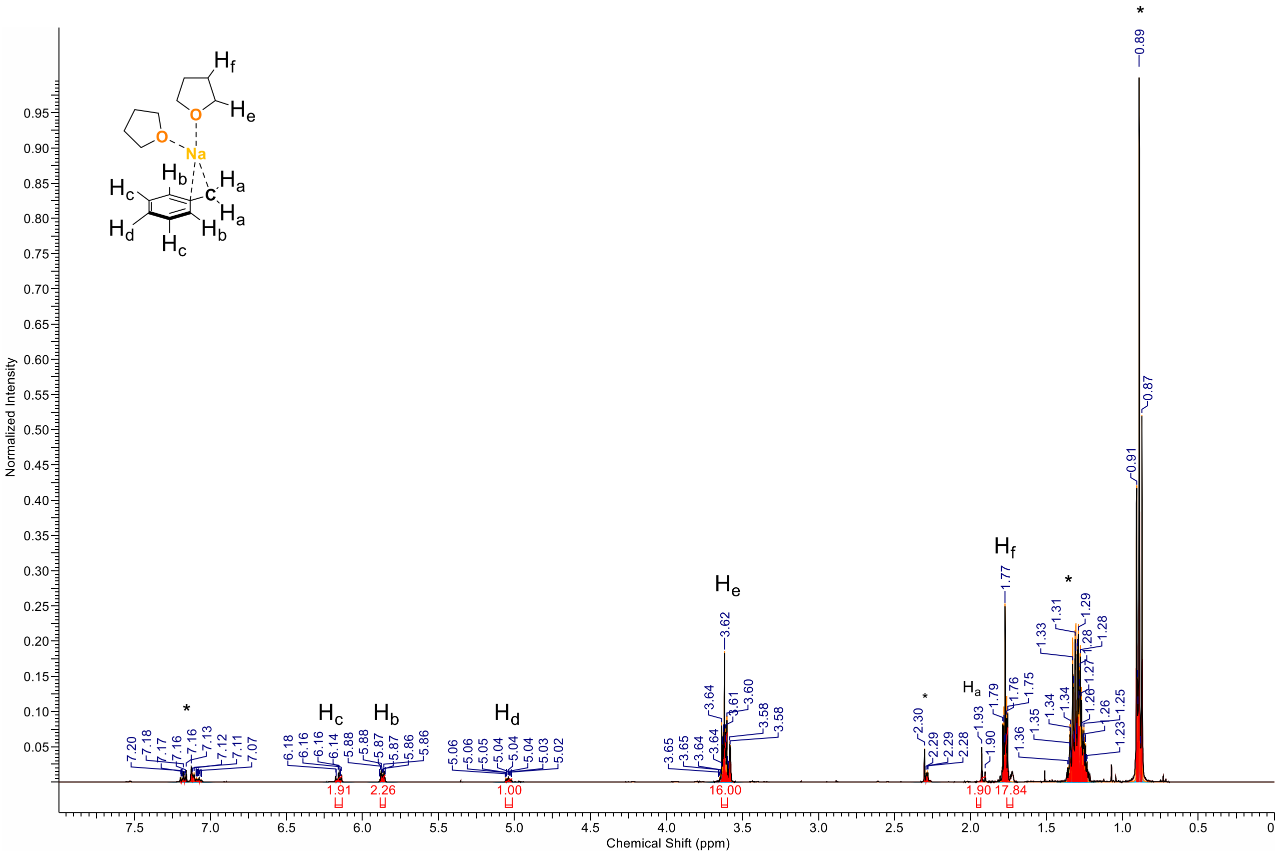

Figure S3 ^{1}H-NMR spectrum of **3a**. "*" indicates impurities due to decomposition reactions or reactants.

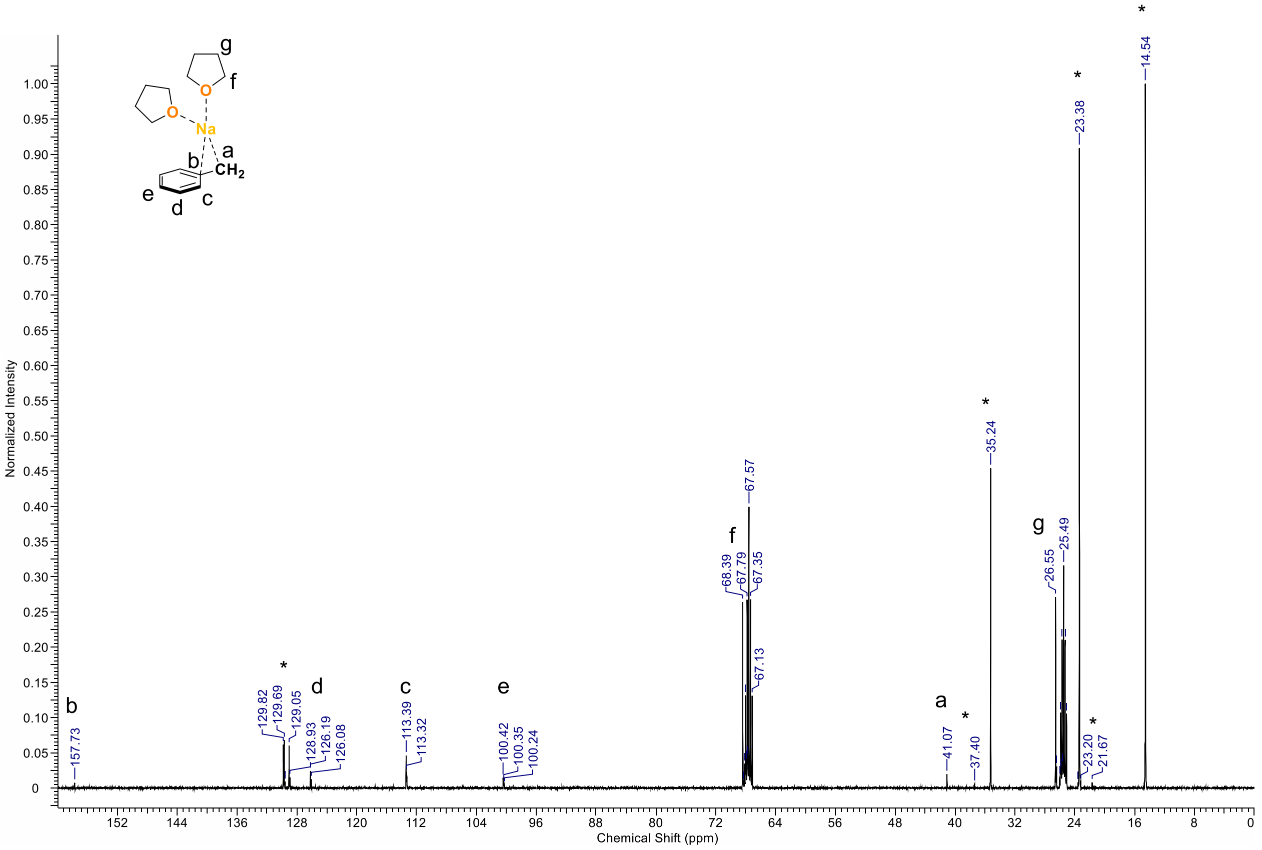

Figure S4 ^{13}C-NMR spectrum of **3a**. “*” indicates impurities due to decomposition reactions or reactants.

3.2 Synthesis of the benzylrubidium thf-solvate 3c

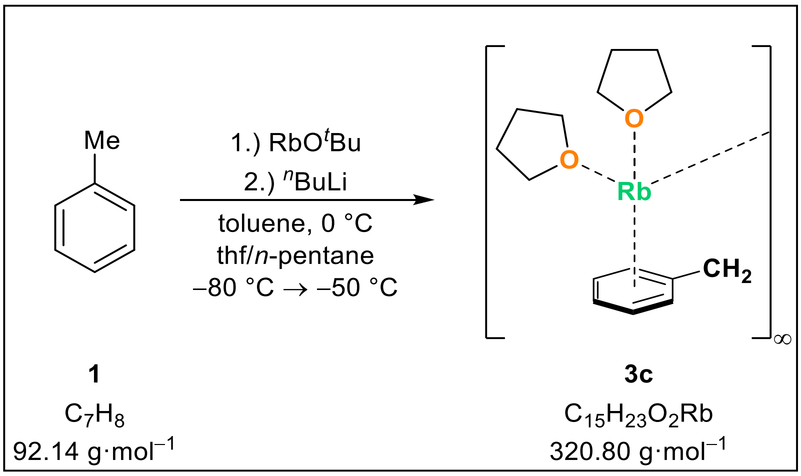

To a stirred solution of toluene (92 mg, 1.0 mmol, 1.0 eq.) and rubidium *tert*-butoxide (158 mg, 1.0 mmol, 1.0 eq.) at 0 °C *n*-butyllithium (0.44 ml, 1.1 mmol, 1.1 eq. 2.5 M in hexane) was added. An orange-red solid precipitated immediately and after a few minutes the remaining solvent was removed, the precipitate was washed with *n*-pentane (3 x 5 ml) and dried in the vacuum. Then the orange-red solid was dissolved in thf, cooled down to –80 °C and layered with *n*-pentane (1.0 ml). After one day at –80 °C red needles of compound **3c** formed, suitable for single crystal X-ray analysis. The mother liquor was removed and the crystals were washed with cold *n*-pentane (3 x 1 ml) until the solvent remained colorless. Due to the weak thf-metal bond the crystals were not weight stable at room temperature, but with a cooled sample the yield of 266 mg (83%, 0.83 mmol) could be estimated.

Nevertheless, a removal of the solvent to the fullest was not accomplished, because we obtained a decomposition of the highly sensitive and reactive compound, causing slight impurities in the NMR spectra. Anyway, the most characteristic signals could be characterized, like the highly shifted protons of the phenyl in the ^{1}H-NMR rings as well as the shifted benzylic and the *ipso*-carbon atom in the ^{13}C-NMR.

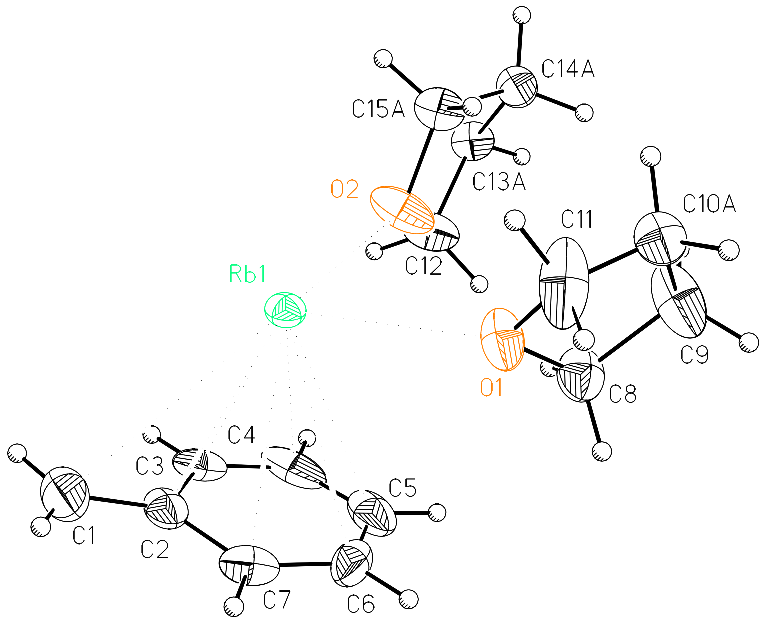

3c

Figure S5 Ortep plot[7] of the asymmetric unit of **3c** in the crystal, with displacement ellipsoids drawn at the 50% probability level. Numbering scheme of hydrogen atoms and disorders are omitted for clarity. Selected bond lengths [Å] and angles [°]: Rb1–O1 2.812(3), Rb1–O2 2.839(3), Rb1–C2 3.105(6), Rb1–C3 3.269(6), Rb1–C6 3.420(6), Rb1–C7 3.230(6), C1–C2 1.357(8), C2–C3 1.442(9), C2–C7 1.446(11), C3–C4 1.335(11), C4–C5 1.362(14), C5–C6 1.390(11), C6–C7 1.395(9); C1–C2–Rb1 100.7(4), C1–C2–C3 122.7(7), C1–C2–C7 123.5(7), C3–C2–C7 113.7(5), C4–C3–C2 123.0(7), C3–C4–C5 123.4(8), C4–C5–C6 117.1(6), C5–C6–C7 122.5(6), C6–C7–C2 120.2(6), O1–Rb1–O2 79.2(10).

^{1}H-NMR (400.25 MHz, thf-d$_8$, 298 K): δ = 1.76-1.79 [m, 4H; β-CH_2 (thf)], 2.25 [s, 2H; C(CH_2)], 3.60-3.64 [m, 4H; α-CH_2 (thf)], 4.70 [tt, $^{3}J_{(H,H)}$ = 6.6 Hz, $^{4}J_{(H,H)}$ = 1.0 Hz, 1H; H_{para}], 5.47 [dd, $^{3}J_{(H,H)}$ = 8.6 Hz, $^{4}J_{(H,H)}$ = 1.2 Hz, 2H; H_{ortho}], 6.02 [dd,$^{3}J_{(H,H)}$ = 8.6 Hz, $^{3}J_{(H,H)}$ = 6.6 Hz ,2H; H_{meta}] ppm.

{^{1}H}^{13}C-NMR (100.64 MHz, thf-d$_8$, 298 K): δ = 26.5 [2C; β-CH$_2$ (thf)] 54.6 (1C, CRbCH$_2$), 68.4 [2C; α-CH$_2$ (thf)], 95.2 (1C; C_{para}), 111.4 (2C; C_{ortho}), 130.9 (2C; C_{meta}), 153.2 (1C; C_{ipso}) ppm.

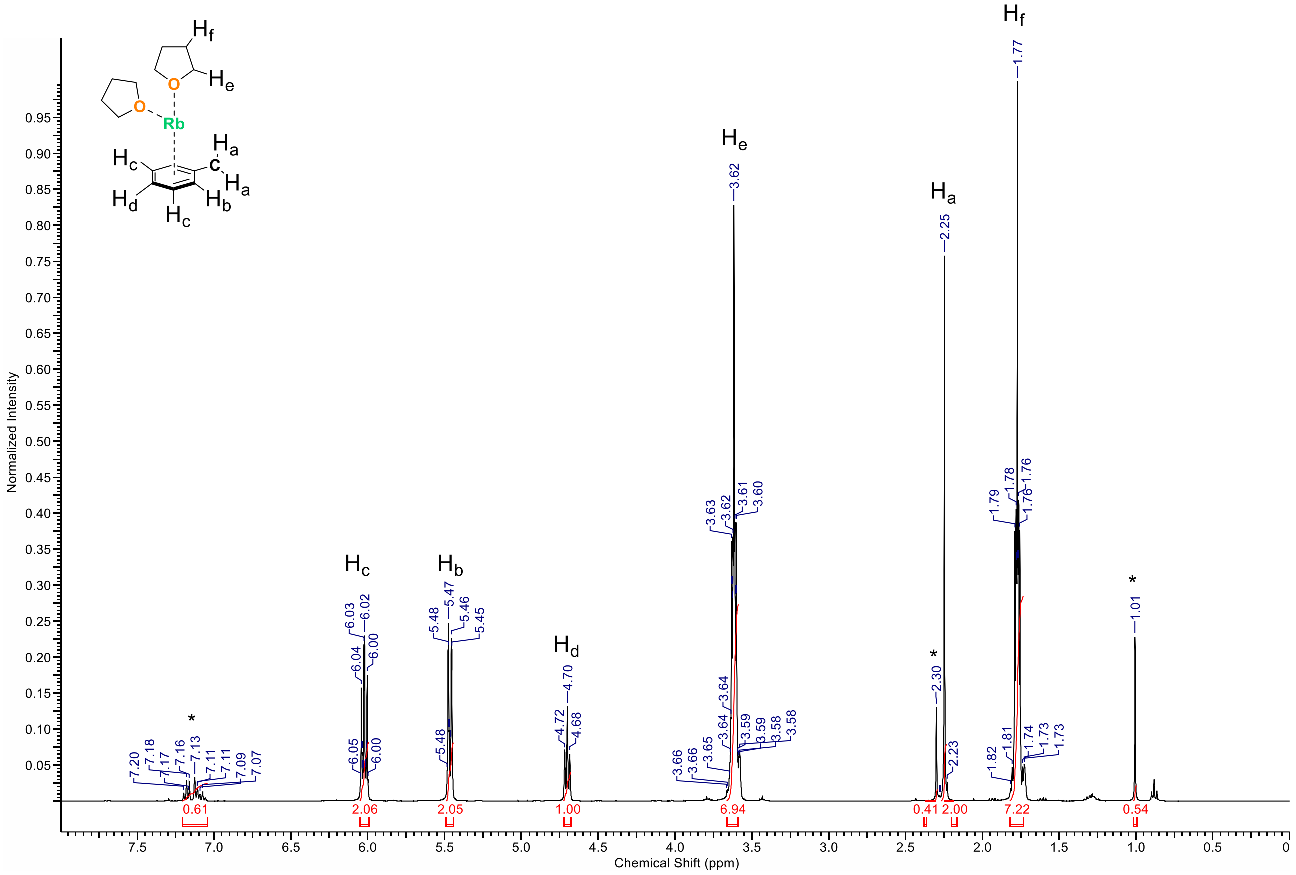

Figure S6 ^{1}H-NMR spectrum of **3c**. "*" indicates impurities due to decomposition reactions or reactants.

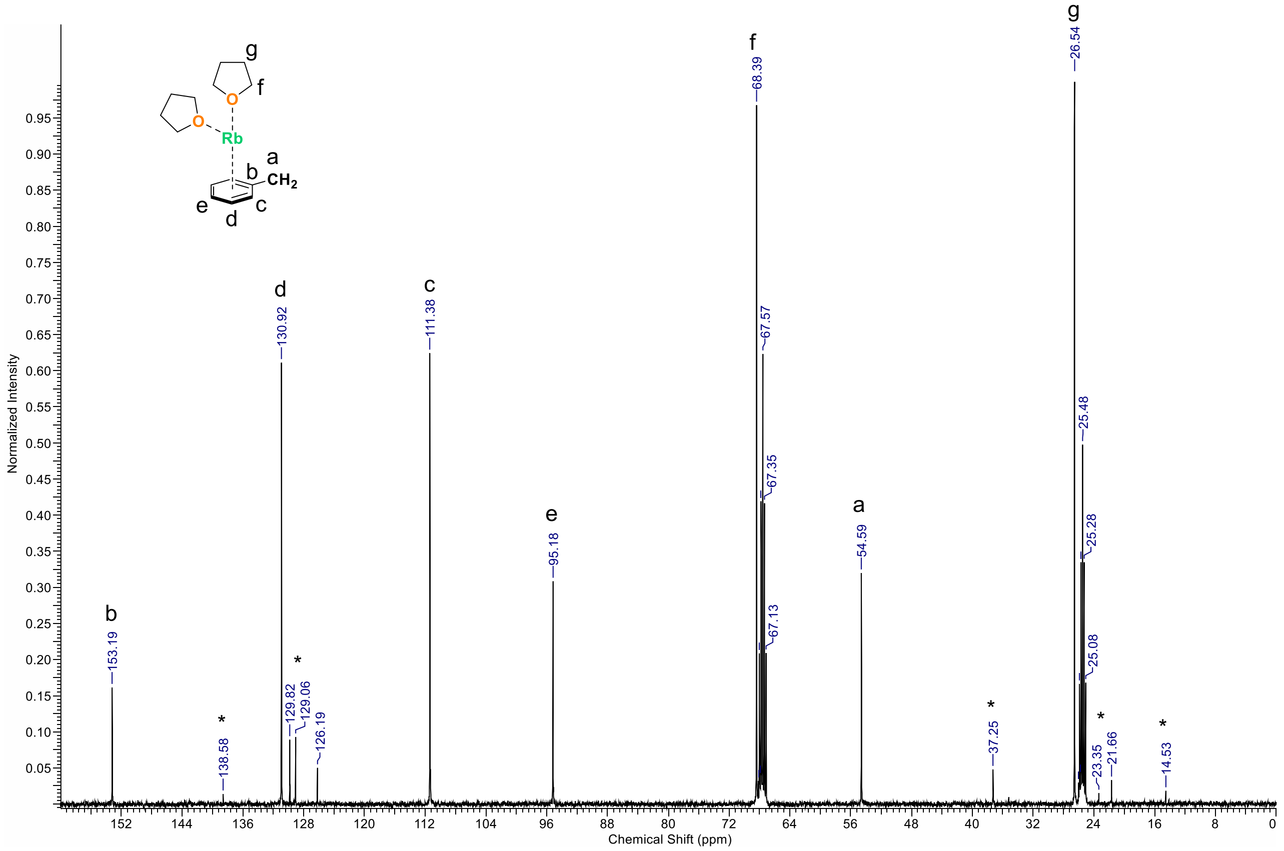

Figure S7 ^{13}C-NMR spectrum of **3c**. "*" indicates impurities due to decomposition reactions or reactants.

3.3 Synthesis of the benzylcaesium thf-solvate 3d

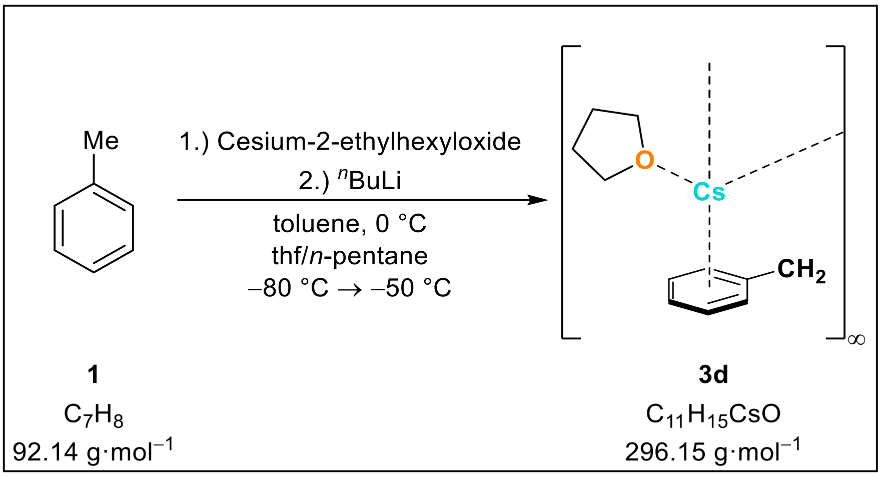

To a stirred solution of toluene (92 mg, 1.0 mmol, 1.0 eq.) and caesium-2-ethylhexyloxide (1.25 ml, 1.0 mmol, 1.0 eq., 0.8 M in octane/toluene) at 0 °C *n*-butyllithium (0.44 ml, 1.1 mmol, 1.1 eq. 2.5 M in hexane) was added. An orange-red solid precipitated immediately and after a few minutes the remaining solvent was removed, the precipitate was washed with *n*-pentane (3 x 5 ml) and dried in the vacuum. Then the orange-red solid was dissolved in thf, cooled down to –80 °C and layered with *n*-pentane (1.0 ml). After storage at –80 °C for one day orange plates of compound **3d** formed. The plates were suitable for single crystal X-ray analysis. The mother liquor was removed and the crystals were washed with cold *n*-pentane (3 x 1 ml) until the solvent remained colorless. Due to the weak thf-metal bond the crystals were not weight stable at room temperature, but with a cold sample a yield of 210 mg (71%, 0.71 mmol) could be estimated. Because of the high sensitivity and reactivity of this compound, neither a complete removal of the very soluble alkoxide, nor the residual solvent was possible, which cause slight impurities in the NMR spectra. Nevertheless, the most characteristic signals could be characterized, like the highly shifted protons of the phenyl in the ^{1}H-NMR rings as well as the shifted benzylic and the *ipso*-carbon atom in the ^{13}C-NMR.

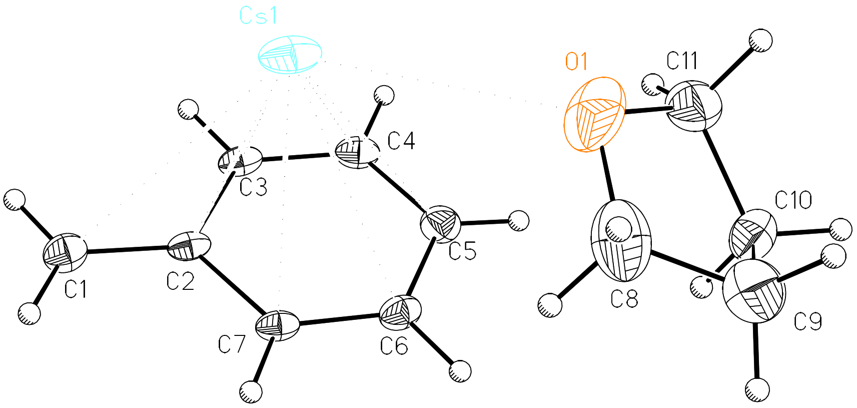

3d

Figure S8 Ortep plot[7] of the asymmetric unit of **3d** in the crystal, with displacement ellipsoids drawn at the 50% probability level. Numbering scheme of hydrogen atoms and disorders are omitted for clarity. Selected bond lengths [Å] and angles [°]: Cs1–O1 3.159(4), Cs1–C2 3.267(2), Cs1–C3 3.432(2), Cs1–C7 3.372(2), C1–C2 1.381(3), C2–C3 1.445(2), C2–C7 1.442(2), C3–C4 1.379(3), C4–C5 1.401(2), C5–C6 1.401(2), C6–C7 1.378(2); C1–C2–Cs1

101.3(1), C1–C2–C3 123.8(2), C1–C2–C7 122.4(2), C3–C2–C7 113.8(2), C4–C3–C2 122.3(2), C3–C4–C5 122.1(2), C4–C5–C6 117.3(2), C5–C6–C7 121.7(2), C6–C7–C2 122.9(2).

^{1}H-NMR (500.1 MHz, thf-d$_8$, 298 K): δ = 1.76-1.79 [m, 4H; β-C*H*$_2$ (thf)], 2.29 [s, 2H; C(C*H*$_2$)], 3.60-3.64 [m, 4H; α-C*H*$_2$ (thf)], 4.68 [tt, $^{3}J_{(H,H)}$ = 6.6 Hz, $^{4}J_{(H,H)}$ = 1.1 Hz, 1H; H_{para}], 5.35 [dd, $^{3}J_{(H,H)}$ = 8.6 Hz, $^{4}J_{(H,H)}$ = 1.3 Hz, 2H; H_{ortho}], 5.96 [dd, $^{3}J_{(H,H)}$ = 8.5 Hz, $^{3}J_{(H,H)}$ = 6.6 Hz, 2H; H_{meta}] ppm.

{^{1}H}^{13}C-NMR (125.74 MHz, thf-d$_8$, 298 K): δ = 26.5 [2C; β-CH$_2$ (thf)], 56.3 (1C, CCsCH$_2$), 68.4 [2C; α-CH$_2$ (thf)], 94.6 (1C; C_{para}), 112.7 (2C; C_{ortho}), 131.0 (2C; C_{meta}), 154.1 (1C; C_{ipso}) ppm.

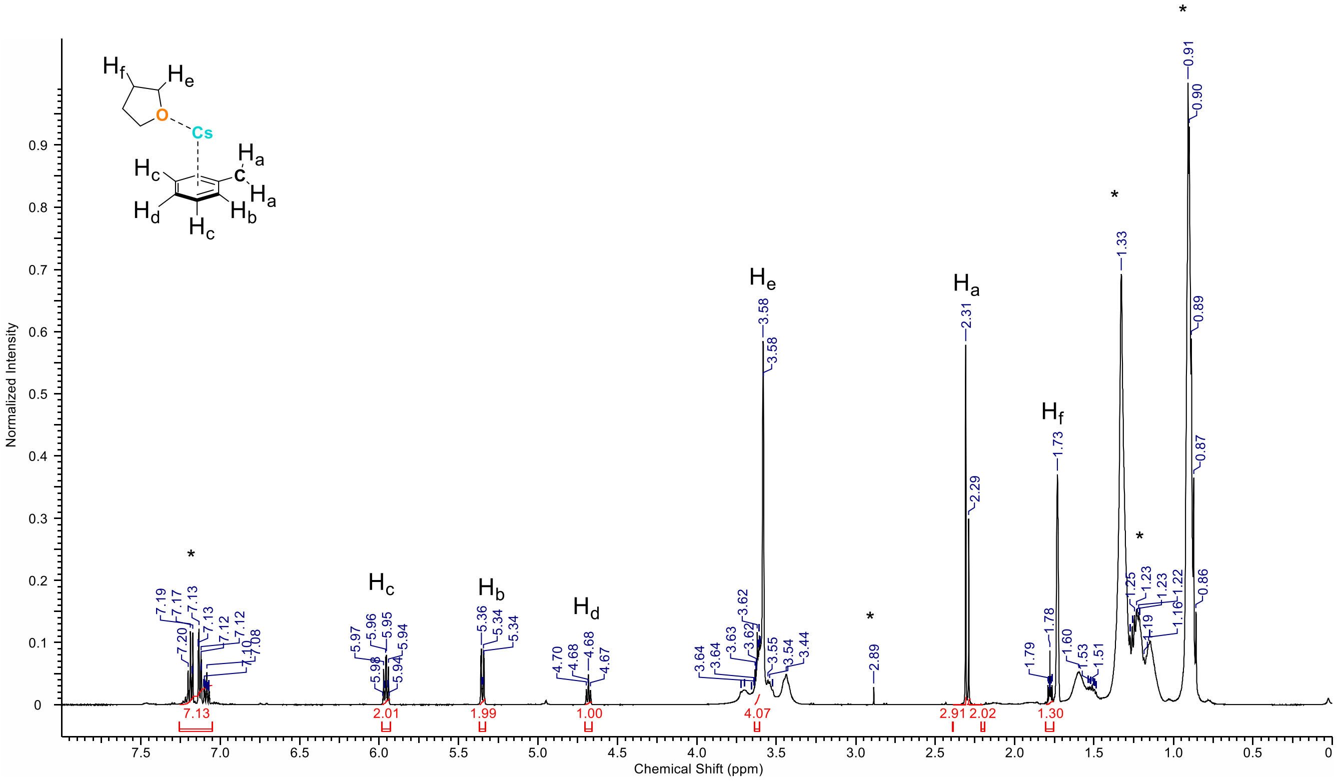

Figure S9 ^{1}H-NMR spectrum of **3d**. “*” indicates impurities due to decomposition reactions or reactants.

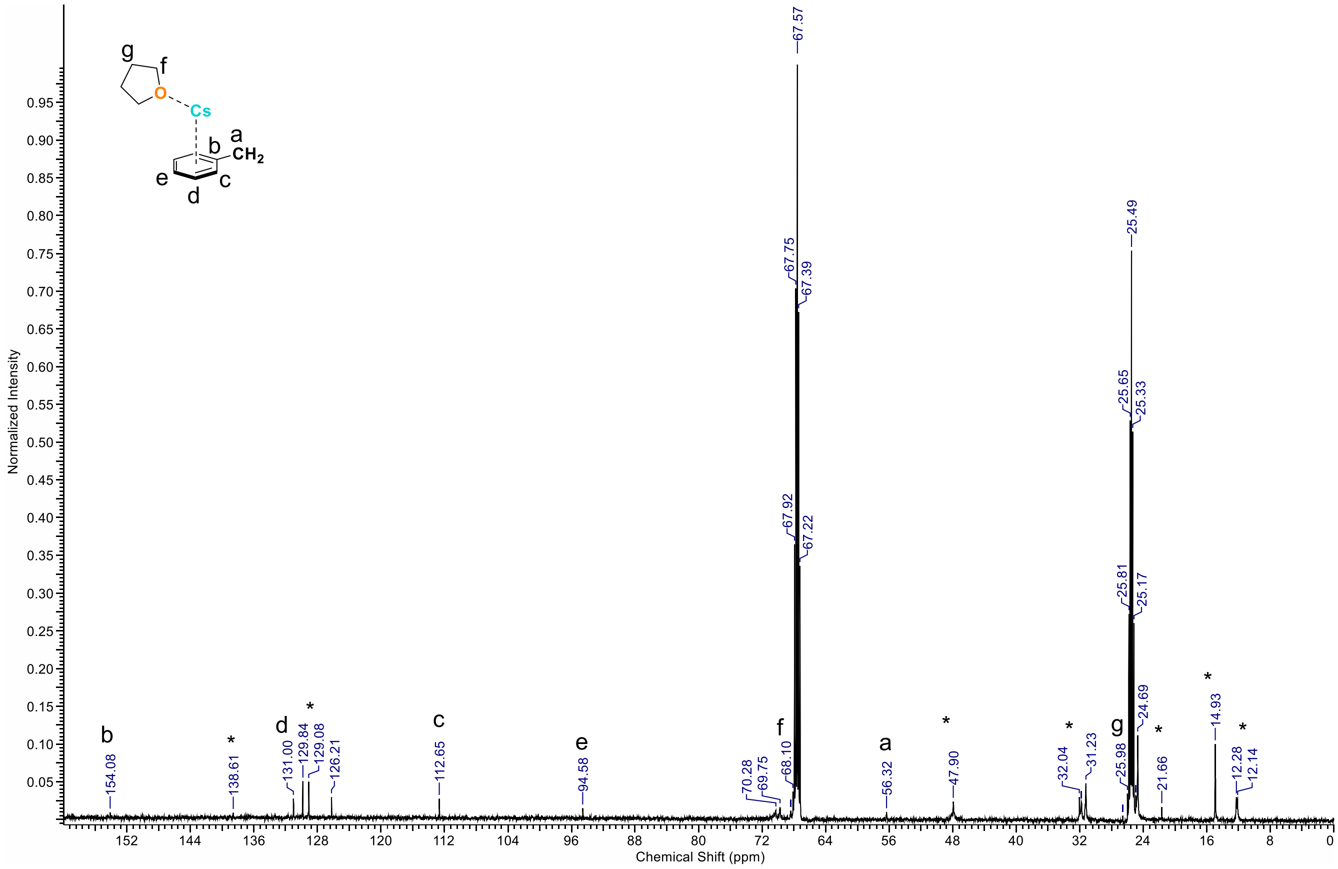

Figure S10 ^{13}C-NMR spectrum of **3d**.

3.4 Synthesis of metalated compound 5

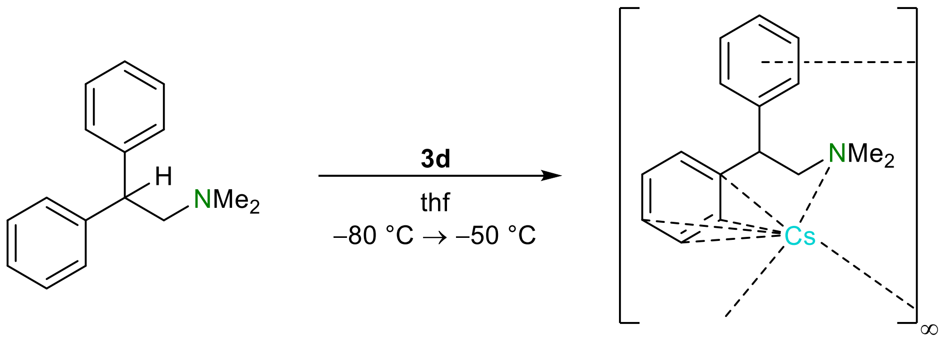

4
$C_{16}H_{19}N$
225.34 g·mol^{-1}

5
$C_{16}H_{18}CsN$
357.22 g·mol^{-}1

To a stirred solution of *N*,*N*-Dimethyl-2,2-diphenyl-ethyl-1-amine (**4**) (245 mg, 1.09 mmol, 1.0 eq.) compound **3d** (371 mg, 1.09 mmol, 1.0 eq) was added at –80 °C. The reaction was stirred for one hour and was allowed to warm up from –80 °C to –50 °C. During this time the color of the mixture turned from red to dark red. After two weeks red blocks of compound **5** formed, suitable for single crystal X-ray analysis. The mother liquor was removed and the crystals were washed with cold *n*-pentane (3 x 1 ml) until the solvent remained colorless. Keeping the red crystals in vacuum to remove the solvent led to slight decomposition of the crystals. Therefore, a yield was not determined. Because of the high sensitivity of the crystals, a complete removal of the washing solvents was not possible, which cause slight impurities in the NMR spectra. Nevertheless, the most characteristic signals could be assigned, like

the highly shifted protons of the phenyl in the ^{1}H-NMR rings as well as the shifted benzylic carbon atom in the ^{13}C-NMR.

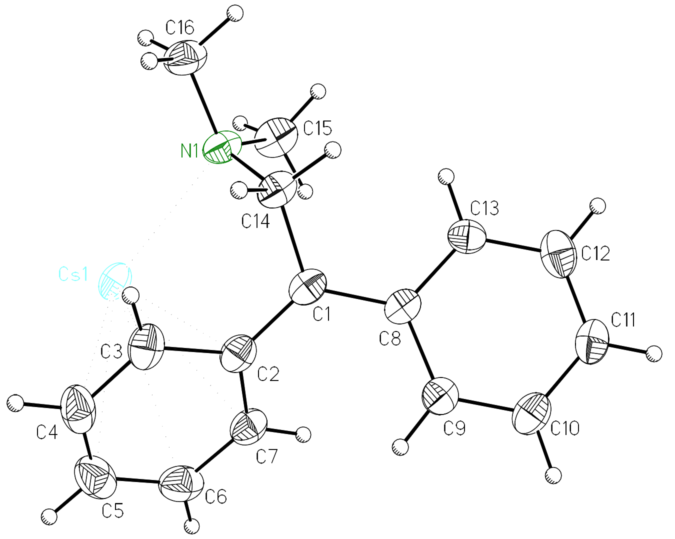

5

Figure S11 Ortep plot[7] of the asymmetric unit of **5** in the crystal, with displacement ellipsoids drawn at the 50% probability level. Numbering scheme of hydrogen atoms and disorders are omitted for clarity. Selected bond lengths [Å] and angles [°]: Cs1–N1 3.370(3), Cs1–C2 3.398(4), Cs1–C3 3.314(4), Cs1–C4 3.401(4), Cs1–C7 3.506(4), C1–C2 1.426(5), C2–C3 1.442(5), C2–C7 1.433(5), C3–C4 1.369(6), C4–C5 1.372(7), C5–C6 1.398(7), C6–C7 1.386(6), C1–C8 1.445(5), C8–C9 1.417(5), C9–C10 1.380(5), C10–C11 1.410(6); C1–C2–Cs1 109.0(2), C1–C2–C3 122.3(3), C1–C2–C7 123.8(3), C3–C2–C7 113.8(3), C4–C3–C2 122.3(4), C3–C4–C5 122.4(4), C4–C5–C6 118.1(4), C5–C6–C7 120.9(4), C6–C7–C2 122.5(4), C1–C8–C9 123.1(3), C8–C9–C10 123.4(4), C9–C10–C11 120.9(4), C10–C11–C12 118.2(4).

^{1}H-NMR (400.25 MHz, thf-d$_8$, 298 K): δ = 2.11 [s, 6H; N(CH_3)$_2$], 3.12 [s, 2H; NCH_2C], 5.84 [t, $^{3}J_{(H,H)}$ = 7.0 Hz, 2H; H_{para}], 6.62 [dd, $^{3}J_{(H,H)}$ = 8.6 Hz, $^{3}J_{(H,H)}$ = 7.0 Hz, 4H; H_{meta}], 6.96 [m, 4H; H_{ortho}] ppm.

{^{1}H}^{13}C-NMR (100.64 MHz, thf-d$_8$, 298 K): 45.9 [2C; N(CH$_3$)$_2$], 63.9 (1C, NCH$_2$$C$), 81.7 (1C; N$CH_2$C) 110.0 (2C; C_{para}), 117.9 (4C; C_{ortho}), 129.4 (4C; C_{meta}), 148.2 (2C; C_{ipso}) ppm.

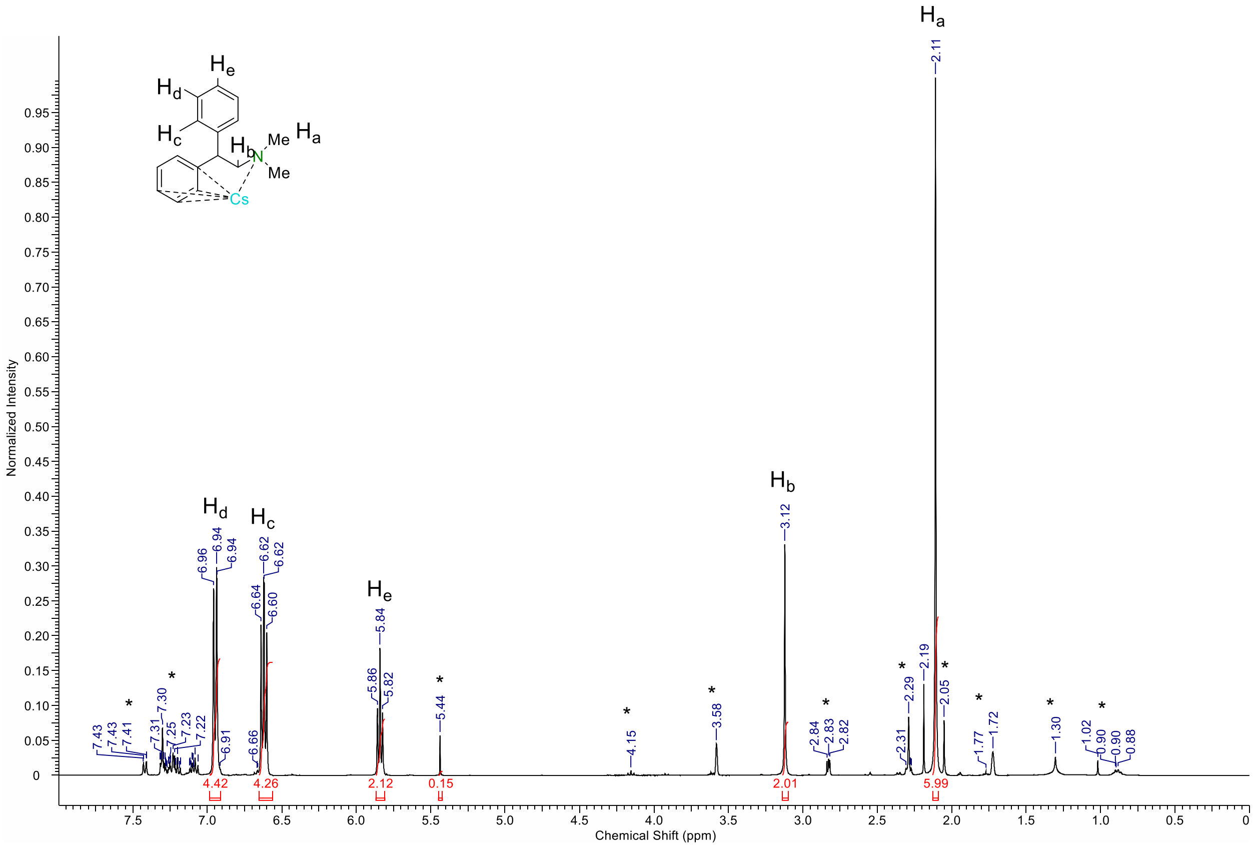

Figure S12 ^{1}H-NMR spectrum of compound **5**. “*” indicates impurities due to decomposition reactions or reactants.

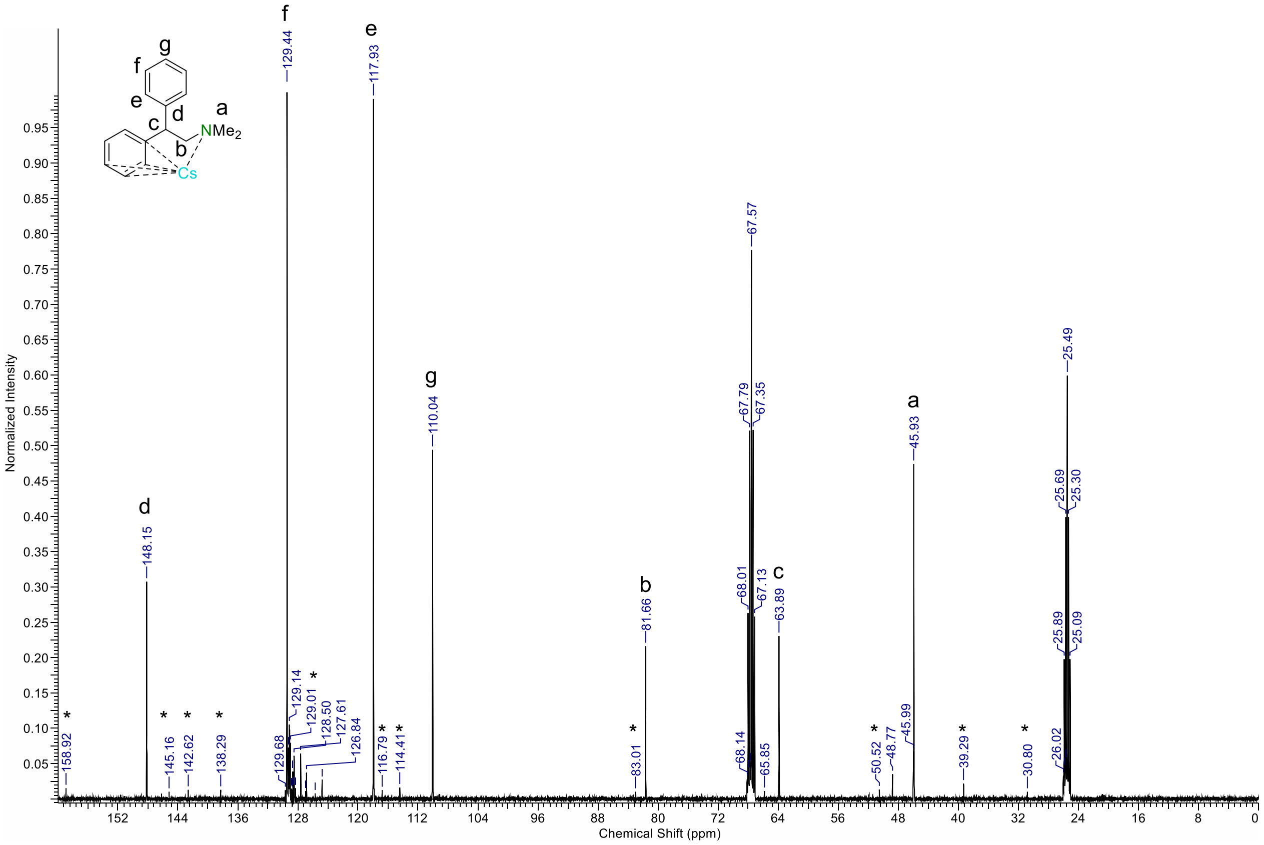

Figure S13 ^{13}C-NMR spectrum of compound **5**. "*" indicates impurities due to decomposition reactions or reactants.

Table S2 Crystal data and structure refinement for **3a**, **3c** and **3d**.

Compound	**[PhCH$_2$Na(thf)$_2$]$_\infty$ 3a**	**[PhCH$_2$Rb(thf)$_2$]$_\infty$ 3c**	**[PhCH$_2$Cs(thf)$_{0.5}$]$_\infty$ 3d**
Empirical formula	$C_{15}H_{23}NaO_2$	$C_{15}H_{23}O_2Rb$	$C_{18}H_{22}Cs_2O$
Formula weight [g·mol^{-1}]	258.32	320.80	520.19
Temperature [K]	100.0	100.0	100.0
Wavelength [Å]	0.71073	0.71073	0.71073
Crystal System	orthorhombic	orthorhombic	monoclinic
Space Group (Nr.)	$P2_12_12_1$	$Pna2_1$	$C2/c$
a [Å]	8.0190(18)	14.9799(6)	9.1835(5)
b [Å]	9.4008(18)	8.6613(5)	8.9470(5)
c [Å]	19.833(8)	11.8341(5)	21.9384(17)
α [°]	90	90	90
β [°]	90	90	93.504(3)
γ [°]	90	90	90
Volume [Å^3]	1495.1(7)	1535.42(13)	1799.2(2)

Z	4	4	4
Density (calculated) *ρ* [g·cm^{-3}]	1.148	1.388	1.920
Absorption coefficient *μ* [mm^{-1}]	0.098	3.218	4.049
F(000)	560.0	664.0	990.5
Crystal size [mm^3]	0.444 × 0.432 × 0.096	0.796 × 0.06 × 0.052	0.281 × 0.227 × 0.062
Theta range for data collection *θ* [°]	4.796 to 57.996	5.432 to 61.068	6.36 to 64.94
Index ranges	–10 ≤ h ≤ 10, –12 ≤ k ≤ 12, –27 ≤ l ≤ 26	–21 ≤ h ≤ 21, –12 ≤ k ≤ 12, –16 ≤ l ≤ 16	–14 ≤ h ≤ 14, –14 ≤ k ≤ 14, –35 ≤ l ≤ 35
Reflections collected	26067	54880	43677
Independent reflections	3965 [R_{int} = 0.0290 R_{sigma} = 0.0196]	4686 [R_{int} = 0.0434, R_{sigma} = 0.0206]	3261 [R_{int} = 0.0401, R_{sigma} = 0.0204]
Structural refinement	Full-matrix least -squares on F^2		
Data / restraints / parameters	3965/0/182	4686/1/190	3261/69/146
Goodness-of-fit an F^2	1.050	1.106	1.061
Final *R* indices [$I > 2σ(I)$]	R_1 = 0.0286, wR_2 = 0.0752	R_1 = 0.0314, wR_2 = 0.0789	R_1 = 0.0207, wR_2 = 0.0451
R indices (all data)	R_1 = 0.0304, wR_2 = 0.0767	R_1 = 0.0375, wR_2 = 0.0813	R_1 = 0.0255, wR_2 = 0.0474
absolute structure parameter	0.00(6)	0.485(15)[a]	–
Largest diff. Peak and hole [e·Å^{-3}]	0.20/–0.15	1.10/–0.53	0.86/–1.03

[a]Twin-Law for the inversion twin (–1, 0, 0, 0, –1, 0, 0, 0, –1) BASF [0.485.(15)]

Table S3 Crystal data and structure refinement for **5**.

Compound	**5**
Empirical formula	$C_{16}H_{18}CsN$
Formula weight [g·mol^{-1}]	357.22
Temperature [K]	100.0
Wavelength [Å]	0.71073
Crystal System	trigonal
Space Group (Nr.)	R-3
a [Å]	24.4211(16)
b [Å]	24.4211(16)
c [Å]	17.805(2)
α [°]	90
β [°]	90
γ [°]	120
Volume [Å^3]	9195.9(16)
Z	18
Density (calculated) ρ [g·cm^{-3}]	1.161
Absorption coefficient μ [mm^{-1}]	1.800
F(000)	3168.0
Crystal size [mm^3]	0.13 × 0.096 × 0.072
Theta range for data collection θ [°]	4.48 to 56
Index ranges	–26 ≤ h ≤ 32, –32 ≤ k ≤ 28, –23 ≤ l ≤ 23
Reflections collected	58818
Independent reflections	4921 [R_{int} = 0.0593, R_{sigma} = 0.0282]
Structural refinement	Full-matrix least -squares on F^2
Data / restraints / parameters	4921/0/165
Goodness-of-fit an F^2	1.052
Final *R* indices [$I > 2\sigma(I)$]	R_1 = 0.0416, wR_2 = 0.1043
R indices (all data)	R_1 = 0.0468, wR_2 = 0.1070
absolute structure parameter	–
Largest diff. Peak and hole [e·Å^{-3}]	0.91/–1.67

3.5 Re-recorded NMR spectra of compound 3b

The ^{1}H- and ^{13}C-NMR spectra of compound 3b were re-recorded, since the old data was accomplished on a 200 MHz spectrometer and the concentration was very low. In the following the spectra will be shown. The NMR spectra were recorded at 298 K.

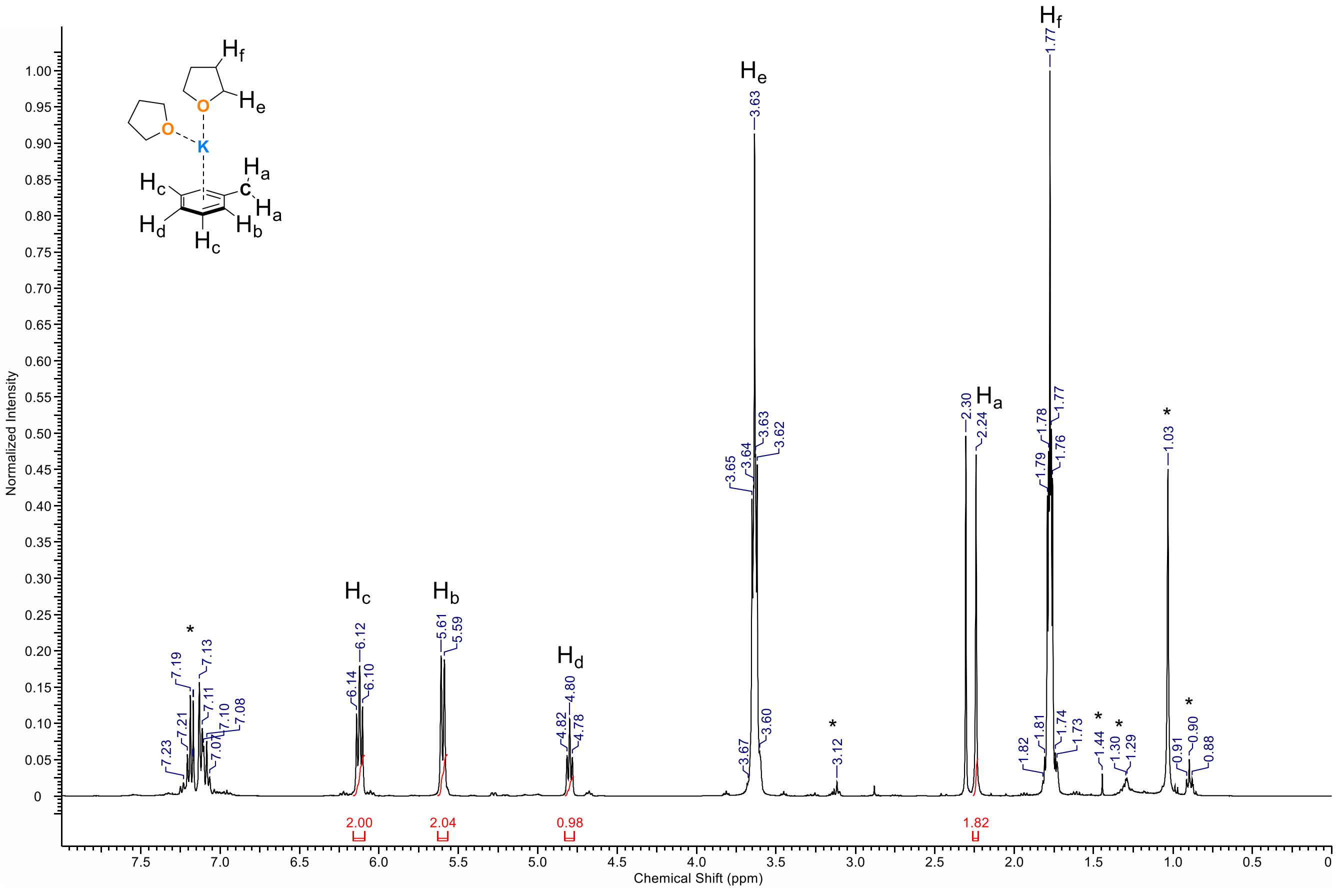

Figure S14 ^{1}H-NMR spectrum of compound **3b**. “*” indicates impurities due to decomposition reactions or reactants.

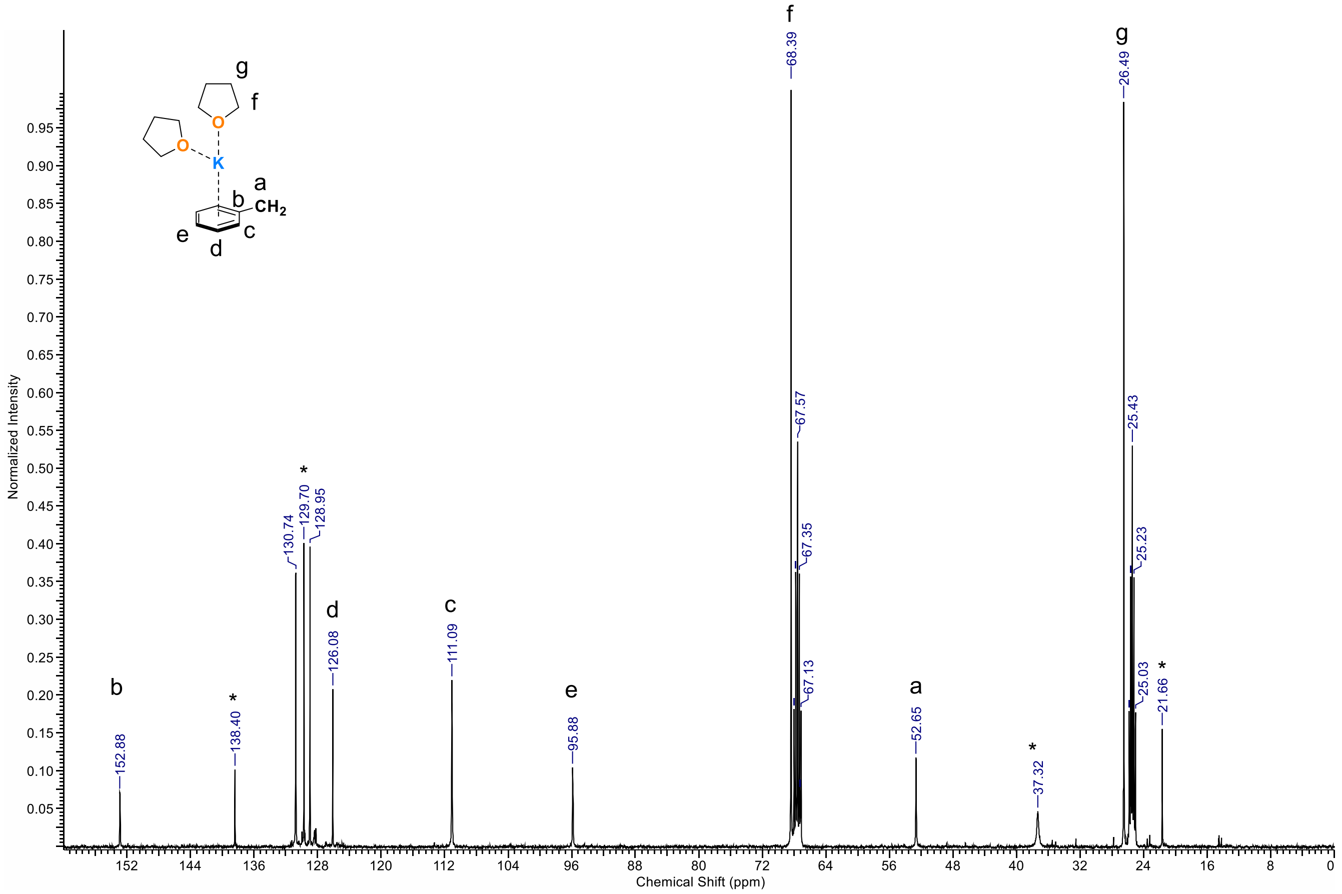

Figure S15 ^{13}C-NMR spectrum of compound **3b**. "*" indicates impurities due to decomposition reactions or reactants.

3.6 Decomposition of the thf-solvated benzyl compounds in thf at room temperature

In the course of further 1H-DOSY NMR studies, we observed the decomposition of the benzyl compound in thf-d_8 within 30 minutes. The decomposition was accompanied by a clear color change of the sample.

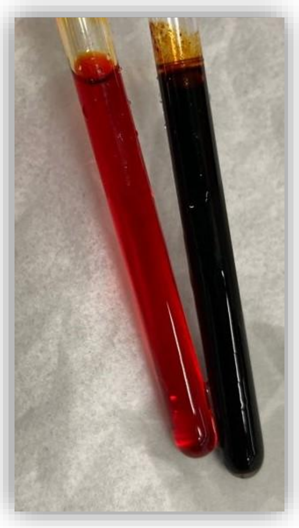

Figure S16 Exemplary presentation of a freshly prepared and cooled sample of the thf-solvated benzylic compound **3b** in thf-d_8 (left) and the sample after 30 minutes of measuring at room temperature (right).

4. Quantum Chemical Calculations

Optimization and additional harmonic vibrational frequency analyses were performed with the software package Gaussian 16 (Revision B.01)[8] on the B3LYP-D3[9]/def2-tzvpp[10] level of theory using Densitiy-Functional Theory (DFT).[11] The basis set used features a triple-zeta representation of valence orbitals and an extended set of polarization functions. For the elements Rb and Cs the electrons of the inner shell are modeled by effective core potentials (ECPs), which reduce the required basis set size and incorporate scalar relativistic effects. The GJF input-files were created with the program GaussView 6.0.[12] The ground state structures were optimized without symmetry restrictions. Vibrational frequency analysis showed no imaginary frequency in the harmonical approximation for the ground states. The calculated standard orientations of the optimized structures can be found in the following Tables. The NBO analyses[13] were perfomed with Gaussian 16. The visualization of the molecular orbitals was done with the MOLDEN[14] and IboView[15] program packages.

4.1 Calculated Monomers of Alkali Metal Benzyl Compounds coordinated by thf

In the following, the energies (Table S4) and the coordinates of the optimized structures for the monomers are listed. The number of coordinating thf molecules was adjusted to the respective metal for the calculation of the monomers (Figure S17).

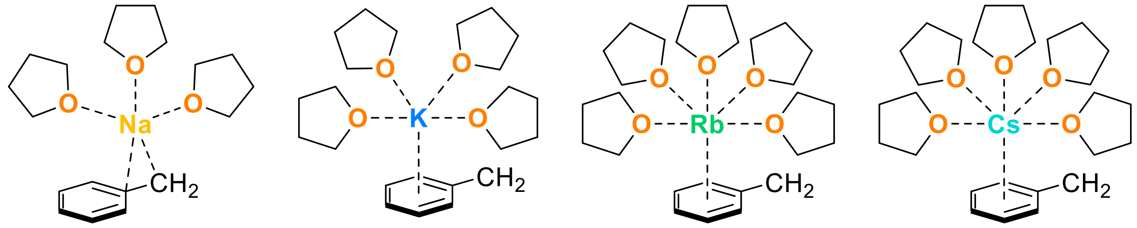

QM1 **QM2** **QM3** **QM4**

Figure S17 Presentation of the calculated monomeric alkali metal benzyl compounds coordinated by thf molecules.

Table S4 Total (SCF) and zero-point-corrected (ZPE) energies of the optimized structures of the calculated alkali metal benzyl monomers coordinated by thf.

Name	Calculation number	SCF [Hartree]	ZPE [Hartree]
**Mono-Na**	QM1	–1131.09923	–1130.629936
**Mono-K**	QM2	–1801.304665	–1800.718282
**Mono-Rb**	QM3	–1458.051061	–1457.346509
**Mono-Cs**	QM4	–1454.102283	–1453.398146

Table S5 Coordinates of **QM1**.

Element	X	Y	Z
**C**	1.732827	0.044247	2.082668
**C**	3.072272	–0.133353	1.786322
**C**	3.581433	–1.379404	1.409839
**C**	2.684759	–2.451424	1.348020
**C**	1.340726	–2.291472	1.630885
**C**	0.781009	–1.028537	2.021507
**H**	1.389469	1.017666	2.415164

**H**	3.743606	0.715032	1.869590
**H**	4.633066	–1.515651	1.199681
**H**	3.049019	–3.437296	1.079908
**H**	0.684520	–3.154778	1.592656
**C**	–0.602302	–0.840952	2.234997
**H**	–1.210047	–1.717543	2.434696
**H**	–0.921244	0.056111	2.756297
**O**	1.286355	–0.029634	–1.959727
**C**	2.509179	–0.802903	–1.985322
**C**	1.601439	1.278573	–2.447918
**C**	3.670333	0.210116	–1.926449
**H**	2.493046	–1.469645	–1.126562
**H**	2.531033	–1.393557	–2.904941
**C**	2.965207	1.577416	–1.841165
**H**	1.646780	1.265619	–3.544362
**H**	0.803370	1.950305	–2.138550
**H**	4.285660	0.145276	–2.823733
**H**	4.303862	0.025932	–1.062231
**H**	3.500134	2.368109	–2.365835
**H**	2.844697	1.872708	–0.798781
**O**	–1.016778	1.884157	–0.240606
**C**	–0.122968	2.842721	0.364398
**C**	–2.323851	1.982360	0.373851
**C**	–0.777789	3.238408	1.679418
**H**	0.849513	2.368803	0.490029
**H**	–0.016631	3.702396	–0.305767
**C**	–2.265917	3.182196	1.317015
**H**	–3.067569	2.091722	–0.416605
**H**	–2.506311	1.053822	0.920079
**H**	–0.455380	4.217950	2.029121
**H**	–0.545857	2.500765	2.447478
**H**	–2.566227	4.095105	0.799383
**H**	–2.913044	3.054044	2.183043
**O**	–2.058480	–1.553941	–1.239840
**C**	–2.613862	–2.569319	–0.390148
**C**	–3.166366	–0.873295	–1.847965
**C**	–3.804948	–1.887901	0.270682
**H**	–2.918456	–3.429126	–0.998754
**H**	–1.840676	–2.876361	0.310686
**C**	–4.338177	–0.961580	–0.844897
**H**	–2.847397	0.145994	–2.061961
**H**	–3.414012	–1.364152	–2.793954
**H**	–3.445130	–1.306826	1.119166
**H**	–4.551732	–2.596195	0.626182
**H**	–4.610396	0.017306	–0.454578
**H**	–5.221637	–1.381125	–1.324521
**Na**	–0.26662	–0.385675	–0.276825

Table S6 Coordinates of **QM2**.

Element	X	Y	Z
**C**	0.377501	1.168342	2.622598
**C**	1.615885	1.720419	2.362975
**C**	2.744304	0.928896	2.096174
**C**	2.574923	–0.462731	2.155362
**C**	1.347637	–1.040371	2.413555
**C**	0.154067	–0.259676	2.649456
**H**	–0.457839	1.822584	2.848241
**H**	1.717700	2.800209	2.380663
**H**	3.716113	1.371712	1.928450
**H**	3.433677	–1.109913	2.012426
**H**	1.278930	–2.119878	2.492068
**C**	–1.098218	–0.829362	2.840504
**H**	–1.192697	–1.898832	2.977899
**H**	–1.925146	–0.227112	3.194880
**O**	0.836742	2.526741	–1.436492
**C**	2.127003	3.035753	–1.035287
**C**	–0.125323	3.578323	–1.303355
**C**	1.870431	4.359082	–0.288346
**H**	2.589820	2.286425	–0.393441
**H**	2.747713	3.178536	–1.923310
**C**	0.346506	4.368606	–0.091724
**H**	–0.130820	4.200743	–2.207762
**H**	–1.102844	3.115058	–1.185824
**H**	2.180158	5.210257	–0.895742
**H**	2.414371	4.404703	0.653005
**H**	–0.071986	5.373400	–0.045844
**H**	0.074947	3.832677	0.818021
**O**	–2.406308	1.073612	–0.717180
**C**	–2.956539	1.422822	0.565269
**C**	–3.437861	1.315546	–1.671584
**C**	–4.435599	1.005054	0.510563
**H**	–2.381615	0.892284	1.322080
**H**	–2.852819	2.503575	0.714704
**C**	–4.721907	0.818200	–1.000904
**H**	–3.496382	2.389717	–1.890896
**H**	–3.178691	0.789805	–2.589614
**H**	–5.075483	1.763571	0.958558
**H**	–4.593433	0.076085	1.054946
**H**	–5.597884	1.370379	–1.338004
**H**	–4.883178	–0.234056	–1.231681
**O**	–1.041765	–2.520667	–0.937258
**C**	–0.789566	–3.442767	0.151317
**C**	–2.458254	–2.417190	–1.142199
**C**	–2.156784	–3.826456	0.731484
**H**	–0.240117	–4.303477	–0.236184
**H**	–0.171709	–2.926733	0.885126

**C**	–3.068238	–2.704134	0.220688
**H**	–2.666633	–1.415982	–1.514676
**H**	–2.781228	–3.150544	–1.891418
**H**	–2.132458	–3.884283	1.817592
**H**	–2.484644	–4.792408	0.343514
**H**	–2.960533	–1.828436	0.862538
**H**	–4.118146	–2.991041	0.167473
**C**	3.623374	–0.789611	–1.329634
**O**	2.298786	–1.117081	–1.799710
**C**	2.231776	–2.535820	–1.989106
**C**	3.081395	–3.098316	–0.858922
**C**	4.254052	–2.104746	–0.827973
**H**	4.196177	–0.348403	–2.149445
**H**	3.516013	–0.052254	–0.534605
**H**	1.183258	–2.825313	–1.959363
**H**	2.647894	–2.804205	–2.968971
**H**	2.519667	–3.055730	0.075062
**H**	3.397178	–4.126815	–1.029877
**H**	4.684063	–1.996379	0.165505
**H**	5.046921	–2.431890	–1.501360
**K**	0.160456	0.009576	–0.407636

Table S7 Coordinates of **QM3**.

Element	X	Y	Z
**C**	0.648785	–1.253152	–3.158154
**C**	1.938369	–1.643385	–2.858915
**C**	2.956615	–0.712671	–2.599482
**C**	2.623443	0.645491	–2.719635
**C**	1.341454	1.060970	–3.017684
**C**	0.251424	0.134790	–3.222280
**H**	–0.094951	–2.011091	–3.380754
**H**	2.170292	–2.703093	–2.841584
**H**	3.971260	–1.028619	–2.400662
**H**	3.396751	1.395934	–2.593450
**H**	1.142226	2.120625	–3.136285
**C**	–1.064214	0.540665	–3.408577
**H**	–1.292955	1.584716	–3.581216
**H**	–1.819630	–0.175979	–3.703921
**O**	1.077002	–2.600918	1.081264
**C**	2.399117	–2.930100	0.598345
**C**	0.204688	–3.716321	0.848943
**C**	2.274998	–4.262063	–0.158405
**H**	2.725385	–2.122999	–0.058772
**H**	3.082992	–2.994806	1.447817
**C**	0.764128	–4.384188	–0.398091
**H**	0.232062	–4.396808	1.709881
**H**	–0.806647	–3.329788	0.732563

**H**	2.627337	–5.090554	0.457899
**H**	2.852543	–4.259629	–1.080688
**H**	0.430586	–5.414282	–0.517651
**H**	0.470797	–3.814911	–1.280998
**O**	–2.472580	–1.680770	–0.021775
**C**	–2.905333	–2.101473	–1.321986
**C**	–3.662003	–1.375773	0.711759
**C**	–4.075932	–1.178261	–1.675971
**H**	–2.060991	–2.008535	–2.001126
**H**	–3.219685	–3.151726	–1.276718
**C**	–4.613283	–0.714638	–0.298279
**H**	–4.092809	–2.304512	1.106686
**H**	–3.379226	–0.735648	1.544015
**H**	–4.830688	–1.692933	–2.268785
**H**	–3.709270	–0.332224	–2.253375
**H**	–5.645449	–1.018141	–0.128302
**H**	–4.570666	0.369822	–0.215366
**O**	–1.080475	2.806005	0.388448
**C**	–0.786757	3.605249	–0.781675
**C**	–2.503590	2.651448	0.503475
**C**	–2.117957	3.826768	–1.512953
**H**	–0.321482	4.541180	–0.464430
**H**	–0.074882	3.050582	–1.393642
**C**	–3.014587	2.729099	–0.927221
**H**	–2.693337	1.703970	1.006461
**H**	–2.917162	3.459506	1.119864
**H**	–2.006156	3.745036	–2.592037
**H**	–2.523501	4.813785	–1.284650
**H**	–2.824038	1.787687	–1.445653
**H**	–4.076948	2.962850	–0.989227
**C**	3.650221	0.947631	0.817635
**O**	2.350797	1.309045	1.336762
**C**	2.247085	2.738623	1.364886
**C**	3.086642	3.200731	0.183750
**C**	4.278970	2.236794	0.261425
**H**	4.246079	0.508382	1.621306
**H**	3.499441	0.201455	0.037255
**H**	1.192266	2.998043	1.298585
**H**	2.647890	3.125126	2.311183
**H**	2.531649	3.046246	–0.742435
**H**	3.377050	4.248647	0.248262
**H**	4.753958	2.072302	–0.703580
**H**	5.034041	2.624932	0.946481
**Rb**	0.024031	–0.025219	–0.048047
**C**	–1.177184	–1.036799	3.434212
**O**	–1.379897	0.170867	2.693679
**C**	–0.802794	1.215025	3.487751
**C**	0.467676	0.609984	4.110161

**C**	0.237538	–0.920556	4.011016
**H**	–1.930339	–1.107995	4.229474
**H**	–1.308826	–1.872850	2.750400
**H**	–0.619715	2.062484	2.830442
**H**	–1.518856	1.516443	4.262026
**H**	1.348385	0.898077	3.541270
**H**	0.599269	0.943926	5.138667
**H**	0.950458	–1.376510	3.326911
**H**	0.322468	–1.422167	4.974278

Table S 8 Coordinates of **QM4**.

Element	X	Y	Z
**C**	1.195234	0.773472	–3.225953
**C**	1.888095	1.892745	–2.808332
**C**	1.230759	3.043301	–2.344350
**C**	–0.171953	3.033328	–2.376575
**C**	–0.886256	1.925206	–2.788623
**C**	–0.246790	0.702514	–3.212146
**H**	1.746491	–0.085188	–3.594976
**H**	2.972263	1.881973	–2.855035
**H**	1.780760	3.926254	–2.050055
**H**	–0.716460	3.925142	–2.084304
**H**	–1.969730	1.974369	–2.819378
**C**	–0.952989	–0.459308	–3.502987
**H**	–2.030817	–0.427471	–3.601021
**H**	–0.454744	–1.302506	–3.963675
**O**	2.659168	1.008192	1.437646
**C**	2.972888	2.354816	1.024669
**C**	3.795923	0.175700	1.179068
**C**	4.251102	2.275252	0.169579
**H**	2.127032	2.732366	0.449553
**H**	3.106339	2.980890	1.910469
**C**	4.418885	0.768544	–0.076736
**H**	4.494040	0.218483	2.025275
**H**	3.442494	–0.847721	1.063846
**H**	5.107788	2.668960	0.717610
**H**	4.151699	2.840592	–0.754822
**H**	5.456521	0.470439	–0.221940
**H**	3.844897	0.464156	–0.952516
**O**	2.064391	–2.586370	–0.069628
**C**	2.662754	–2.657216	–1.369642
**C**	1.337412	–3.810962	0.086421
**C**	1.600055	–3.296623	–2.273677
**H**	2.941019	–1.648032	–1.667765
**H**	3.569854	–3.271784	–1.319074
**C**	0.703719	–4.085713	–1.285207
**H**	2.032987	–4.611468	0.367019

**H**	0.618488	–3.670018	0.890431
**H**	2.050828	–3.935025	–3.031914
**H**	1.019033	–2.530204	–2.782295
**H**	0.672759	–5.151953	–1.504855
**H**	–0.318677	–3.713153	–1.320626
**O**	–3.049011	–1.020249	0.262466
**C**	–3.779120	–0.450026	–0.849190
**C**	–2.999762	–2.449845	0.117106
**C**	–4.219126	–1.621086	–1.730745
**H**	–4.617505	0.129050	–0.457823
**H**	–3.108207	0.219773	–1.390180
**C**	–3.196196	–2.704151	–1.370565
**H**	–2.045343	–2.795395	0.515432
**H**	–3.802110	–2.906632	0.708763
**H**	–4.205936	–1.361748	–2.787402
**H**	–5.228041	–1.944862	–1.468191
**H**	–2.267863	–2.518151	–1.914006
**H**	–3.541331	–3.714715	–1.585957
**Cs**	0.028836	–0.044219	–0.051926
**C**	–0.948677	3.570964	1.262077
**O**	–1.344965	2.254735	1.704718
**C**	–2.774872	2.159395	1.657043
**C**	–3.177450	3.074690	0.510358
**C**	–2.211396	4.250655	0.707341
**H**	–0.516738	4.116828	2.103859
**H**	–0.184592	3.449155	0.493311
**H**	–3.036303	1.112671	1.511953
**H**	–3.205352	2.502942	2.606508
**H**	–2.984617	2.579734	–0.442300
**H**	–4.225634	3.369151	0.544640
**H**	–2.011173	4.793787	–0.213980
**H**	–2.619141	4.956381	1.432510
**C**	0.865367	–1.976390	3.264701
**O**	–0.347451	–2.015659	2.504255
**C**	–1.354965	–1.445337	3.348042
**C**	–0.669330	–0.276561	4.073808
**C**	0.839325	–0.633563	4.009312
**H**	0.881744	–2.820380	3.965604
**H**	1.695796	–2.083355	2.569046
**H**	–2.188425	–1.149879	2.713352
**H**	–1.703561	–2.204458	4.059158
**H**	–0.861925	0.663338	3.560168
**H**	–1.030491	–0.179958	5.097003
**H**	1.392934	0.120553	3.452708
**H**	1.290118	–0.718917	4.997329

4.2 Calculated Dimers of Alkali Metal Benzyl Compounds coordinated by thf

In the following, the energies (Table S9) and the coordinates of the optimized structures for the dimers are listed. The number of coordinating THF molecules was kept the same for the dimer to illustrate the differences between isolated and sandwiched benzyl moieties (**Fehler! Verweisquelle konnte nicht gefunden werden.**).

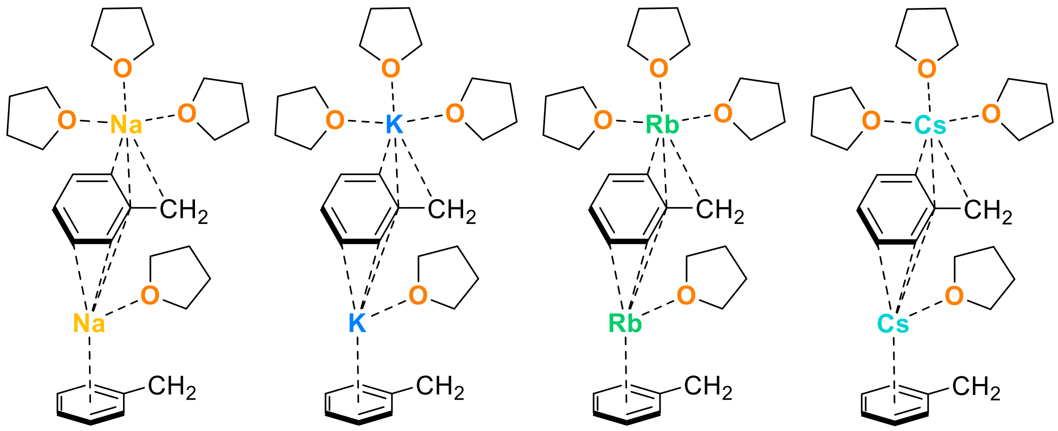

QM5 QM6 QM7 QM8

Figure S18 Presentation of the calculated dimeric alkali metal benzyl compounds coordinated by thf molecules.

Table S9 Total (SCF) and zero-point-corrected (ZPE) energies of the optimized structures of the calculated alkali metal benzyl dimers coordinated by thf.

Name	Calculation number	SCF [Hartree]	ZPE [Hartree]
**Dim–Na**	QM5	–1797.076167	–1796.372458
**Dim–K**	QM6	–2672.343822	–2671.641307
**Dim–Rb**	QM7	–1520.679209	–1519.97829
**Dim–Cs**	QM8	–1512.791307	–1512.090519

Table S10 Coordinates of **QM5**.

Element	X	Y	Z
**O**	2.993793	1.531454	–1.435149
**C**	–0.796815	0.642283	–2.762995
**H**	–0.999174	1.613223	–3.200364
**C**	3.254713	2.129030	–0.141545
**H**	4.334685	2.123413	0.022722
**H**	2.782978	1.514625	0.627296
**O**	–2.143011	–1.832169	1.598246
**C**	4.284715	–2.724049	–1.181505
**H**	4.704360	–3.194291	–2.058668
**C**	3.164490	–3.266839	–0.522994
**H**	2.690367	–4.153916	–0.928886
**C**	–0.428824	0.586801	–1.374249
**C**	–0.103170	–0.735931	–0.904922
**H**	0.248159	–0.856576	0.116485
**C**	2.629946	–0.870832	2.312541

**H**	1.839830	–1.350220	2.874457
**H**	3.131356	–0.039996	2.791738
**C**	3.206003	–1.512558	1.230097
**C**	4.400416	–1.031139	0.569868
**H**	4.916572	–0.183216	1.002972
**O**	–4.053593	0.304785	–0.377766
**C**	–1.036634	–3.911996	1.885606
**H**	–0.679880	–4.586531	2.661666
**H**	–0.377452	–4.010207	1.022478
**C**	4.906052	–1.616312	–0.573571
**H**	5.807243	–1.204958	–1.015109
**C**	2.645884	–2.700598	0.623124
**H**	1.783919	–3.156644	1.094591
**C**	–2.490584	–4.164177	1.472688
**H**	–2.603005	–4.994897	0.777705
**H**	–3.109719	–4.363272	2.349382
**C**	–0.244779	–1.864028	–1.704995
**H**	0.000486	–2.831797	–1.283269
**C**	–0.908935	–0.486995	–3.544406
**C**	–1.080311	–2.460914	2.353179
**H**	–1.324809	–2.382794	3.415561
**H**	–0.150950	–1.925013	2.167123
**C**	–0.661349	–1.770977	–3.032632
**H**	–0.755091	–2.649853	–3.653716
**C**	2.701756	3.547387	–0.219820
**H**	1.649399	3.554895	0.061309
**H**	3.243727	4.236297	0.426886
**C**	–4.427922	0.409887	–2.741889
**H**	–3.810498	0.234688	–3.619732
**H**	–5.471657	0.249063	–3.015950
**C**	–0.425171	1.709332	–0.540629
**C**	2.848799	3.875806	–1.710319
**H**	3.872439	4.181146	–1.935345
**H**	2.176100	4.664506	–2.044580
**C**	–4.239813	1.815431	–2.156638
**H**	–4.860591	2.568611	–2.640162
**H**	–3.195347	2.118253	–2.224249
**C**	2.534262	2.534205	–2.363666
**H**	1.460962	2.402754	–2.511730
**H**	3.053013	2.380767	–3.310611
**C**	–4.606008	1.592008	–0.698800
**H**	–4.171428	2.325098	–0.018586
**H**	–5.692048	1.575000	–0.551104
**H**	0.111212	1.663591	0.399932
**H**	–0.558489	2.695632	–0.965989
**H**	–1.193160	–0.376658	–4.585113
**C**	–2.866321	–2.828334	0.845806
**H**	–2.549188	–2.779942	–0.199452

**H**	–3.927625	–2.589582	0.906527
**C**	–4.021158	–0.507634	–1.582379
**H**	–4.704270	–1.348569	–1.452651
**H**	–3.007919	–0.886828	–1.705956
**C**	–0.159353	1.209865	3.378773
**O**	–1.436872	1.548690	2.783329
**C**	–1.577605	2.983951	2.707536
**C**	–0.426138	3.567029	3.522789
**C**	0.656269	2.494743	3.362368
**H**	–0.335239	0.853409	4.397606
**H**	0.310195	0.416508	2.798147
**H**	–1.512763	3.280078	1.656239
**H**	–2.561736	3.252817	3.093807
**H**	–0.120224	4.547299	3.160038
**H**	–0.711111	3.666053	4.571765
**H**	1.165288	2.599860	2.403483
**H**	1.409196	2.516261	4.147988
**Na**	–2.078792	0.359259	0.857373
**Na**	2.480142	–0.6917	–1.367783

Table S11 Coordinates of **QM6**.

Element	X	Y	Z
**K**	2.526057	–0.898261	–2.040175
**K**	–2.571535	0.743060	1.041405
**O**	2.904659	1.766555	–1.875369
**C**	–1.358518	–0.054762	–2.591401
**H**	–1.872930	0.371511	–3.445014
**C**	3.170322	2.218518	–0.529833
**H**	4.227122	2.491402	–0.453942
**H**	2.970080	1.397963	0.161431
**O**	–2.254871	–1.758792	2.156775
**C**	4.860889	–2.855172	–1.336831
**H**	5.370550	–3.374703	–2.135715
**C**	3.779128	–3.440686	–0.655292
**H**	3.423203	–4.418293	–0.963583
**C**	–0.935693	0.833513	–1.534844
**C**	–0.146076	0.183928	–0.515451
**H**	0.319062	0.789274	0.254581
**C**	2.899047	–0.837341	1.898955
**H**	2.129193	–1.336816	2.473511
**H**	3.306383	0.074164	2.316661
**C**	3.566356	–1.513369	0.890647
**C**	4.716164	–0.970607	0.202156
**H**	5.120150	–0.025550	0.546010
**O**	–4.840690	0.085865	–0.385824
**C**	–0.737602	–3.570640	2.425809
**H**	–0.229390	–4.138349	3.203079

**H**	–0.101272	–3.562115	1.540907
**C**	5.327398	–1.620062	–0.851100
**H**	6.194739	–1.160613	–1.313768
**C**	3.153998	–2.809890	0.401169
**H**	2.333145	–3.309746	0.903243
**C**	–2.125691	–4.117149	2.075245
**H**	–2.093318	–4.968176	1.396552
**H**	–2.660774	–4.416915	2.978359
**C**	0.013837	–1.188889	–0.460140
**H**	0.603504	–1.595200	0.352168
**C**	–1.164704	–1.418598	–2.532495
**C**	–1.052403	–2.142604	2.862546
**H**	–1.251971	–2.081241	3.935959
**H**	–0.257133	–1.443392	2.610651
**C**	–0.505041	–2.031846	–1.451172
**H**	–0.367434	–3.102979	–1.406852
**C**	2.262608	3.424337	–0.318980
**H**	1.276207	3.092789	0.002748
**H**	2.655538	4.118797	0.422859
**C**	–4.972091	–0.557627	–2.691576
**H**	–4.236205	–0.916795	–3.407702
**H**	–5.956900	–0.901261	–3.011160
**C**	–1.297327	2.173515	–1.474544
**C**	2.177194	4.020900	–1.726892
**H**	3.060506	4.626442	–1.938774
**H**	1.291059	4.635321	–1.878151
**C**	–4.955592	0.970321	–2.547514
**H**	–5.575861	1.477096	–3.285697
**H**	–3.937350	1.351487	–2.616676
**C**	2.155333	2.768162	–2.598381
**H**	1.131355	2.412206	–2.733704
**H**	2.628233	2.912974	–3.571103
**C**	–5.458011	1.150126	–1.124479
**H**	–5.158718	2.095176	–0.670483
**H**	–6.549622	1.061529	–1.064557
**H**	–0.857126	2.833706	–0.737893
**H**	–1.820184	2.644918	–2.295770
**H**	–1.531763	–2.033244	–3.348045
**C**	–2.783329	–2.896625	1.444530
**H**	–2.511391	–2.811491	0.388602
**H**	–3.869282	–2.879938	1.540975
**C**	–4.658565	–1.047511	–1.269641
**H**	–5.320267	–1.855324	–0.949823
**H**	–3.624621	–1.378869	–1.180590
**C**	0.379868	1.447575	3.212307
**O**	–0.770485	2.106179	2.626938
**C**	–0.544376	3.526834	2.562128
**C**	0.677039	3.800674	3.435247

**C**	1.476209	2.502446	3.284921
**H**	0.099179	1.081895	4.203970
**H**	0.667024	0.605632	2.584278
**H**	–0.362263	3.809637	1.521377
**H**	–1.445874	4.035790	2.906873
**H**	1.225096	4.684940	3.112807
**H**	0.376517	3.946498	4.474384
**H**	2.048514	2.504054	2.357894
**H**	2.168235	2.321341	4.105043

Table S12 Coordinates of **QM7**.

Element	X	Y	Z
**Rb**	–2.756011	1.295983	–1.319973
**Rb**	2.870436	0.037102	1.297230
**O**	–3.862255	–1.380352	–2.031131
**C**	1.267837	1.787152	–1.290735
**H**	1.678710	1.690183	–2.289940
**C**	–5.280697	–1.630046	–2.033937
**H**	–5.572973	–1.978308	–3.030138
**H**	–5.797945	–0.694673	–1.818922
**O**	5.347826	1.419408	0.543180
**C**	–5.350706	3.292366	0.184657
**H**	–5.768313	4.176652	–0.275364
**C**	–4.230569	3.366257	1.029481
**H**	–3.761212	4.328053	1.208341
**C**	0.423488	0.724950	–0.793519
**C**	–0.112935	0.968510	0.528331
**H**	–0.797893	0.243289	0.956857
**C**	–3.641309	–0.223992	1.943087
**H**	–2.835000	–0.139523	2.659999
**H**	–4.149819	–1.179294	1.893018
**C**	–4.255247	0.926686	1.455661
**C**	–5.457205	0.906324	0.658542
**H**	–5.978336	–0.036485	0.542581
**O**	3.913225	–1.827262	–0.718606
**C**	5.221882	2.581176	–1.471367
**H**	5.445718	3.505805	–2.001422
**H**	4.251881	2.215856	–1.809231
**C**	–5.965096	2.036921	0.048332
**H**	–6.871579	1.949633	–0.542341
**C**	–3.706098	2.247293	1.645997
**H**	–2.838941	2.353701	2.287449
**C**	6.309097	1.506425	–1.650285
**H**	6.109888	0.856094	–2.499928
**H**	7.283528	1.967471	–1.810505
**C**	0.168903	2.122110	1.239073
**H**	–0.293914	2.255561	2.210361

**C**	1.536405	2.924893	–0.555964
**C**	5.189070	2.752587	0.039869
**H**	6.012104	3.387108	0.391743
**H**	4.248512	3.146374	0.421508
**C**	1.014029	3.119799	0.733582
**H**	1.204154	4.028517	1.286080
**C**	–5.524833	–2.700593	–0.974067
**H**	–5.654754	–2.237878	0.004096
**H**	–6.403263	–3.307570	–1.189105
**C**	3.040209	–2.559554	–2.818718
**H**	2.285247	–2.233282	–3.531417
**H**	3.802480	–3.122985	–3.359709
**C**	0.137392	–0.417655	–1.528185
**C**	–4.212274	–3.490365	–1.009996
**H**	–4.210828	–4.195271	–1.844124
**H**	–4.021228	–4.044622	–0.091969
**C**	2.455446	–3.402563	–1.679869
**H**	2.324212	–4.451312	–1.943370
**H**	1.496101	–2.986955	–1.371768
**C**	–3.190130	–2.382584	–1.237823
**H**	–2.888836	–1.929381	–0.288106
**H**	–2.304272	–2.707784	–1.783478
**C**	3.487966	–3.195706	–0.581881
**H**	3.094142	–3.327516	0.426162
**H**	4.350040	–3.859903	–0.711654
**H**	–0.514105	–1.186781	–1.140294
**H**	0.520540	–0.550841	–2.530416
**H**	2.163955	3.692205	–0.996700
**C**	6.288004	0.736868	–0.310769
**H**	5.945916	–0.292351	–0.412374
**H**	7.271656	0.735766	0.166626
**C**	3.665724	–1.378228	–2.072730
**H**	4.611547	–1.054909	–2.510949
**H**	2.980576	–0.530592	–2.026880
**C**	0.946143	–2.218877	3.749913
**O**	1.476476	–2.306400	2.419833
**C**	0.408689	–2.740412	1.538149
**C**	–0.908822	–2.577327	2.314457
**C**	–0.494927	–1.775274	3.556451
**H**	1.002818	–3.197600	4.242074
**H**	1.562880	–1.516543	4.312504
**H**	0.450053	–2.117650	0.644879
**H**	0.591628	–3.777843	1.247096
**H**	–1.672236	–2.056186	1.738980
**H**	–1.306085	–3.550079	2.606156
**H**	–0.531661	–0.706103	3.344493
**H**	–1.127601	–1.972195	4.419981

Table S13 Coordinates of **QM8**.

Element	X	Y	Z
**Cs**	–2.768859	1.635267	–1.143013
**Cs**	2.796784	–0.332048	1.415963
**O**	–3.849162	–1.101007	–2.307371
**C**	1.219993	1.900807	–1.205784
**H**	1.596570	1.735719	–2.209624
**C**	–5.247446	–1.437309	–2.226866
**H**	–5.622889	–1.590527	–3.243208
**H**	–5.786144	–0.603749	–1.773567
**O**	5.265043	1.445807	0.709192
**C**	–5.338496	2.920706	1.109545
**H**	–5.844732	3.852283	0.900617
**C**	–4.161306	2.882742	1.874992
**H**	–3.735754	3.810572	2.242915
**C**	0.475402	0.841843	–0.571434
**C**	–0.035017	1.179773	0.737150
**H**	–0.663263	0.455681	1.247371
**C**	–3.311642	–0.769685	1.916225
**H**	–2.437814	–0.787253	2.553877
**H**	–3.757589	–1.723514	1.664151
**C**	–4.012090	0.414993	1.725446
**C**	–5.261745	0.489021	1.008051
**H**	–5.730020	–0.437959	0.699032
**O**	4.137947	–1.722049	–1.081342
**C**	5.121392	2.766734	–1.206586
**H**	5.255228	3.757823	–1.637872
**H**	4.231390	2.316759	–1.647042
**C**	–5.882180	1.687706	0.715288
**H**	–6.821729	1.670110	0.172098
**C**	–3.524108	1.695921	2.176613
**H**	–2.618221	1.717369	2.771476
**C**	6.349538	1.860491	–1.386749
**H**	6.329792	1.308415	–2.324845
**H**	7.264787	2.452770	–1.373056
**C**	0.207481	2.407177	1.330886
**H**	–0.223621	2.604804	2.305642
**C**	1.449860	3.114273	–0.588668
**C**	4.969341	2.790283	0.306418
**H**	5.680206	3.485919	0.769937
**H**	3.963050	3.032312	0.644636
**C**	0.968287	3.396655	0.698607
**H**	1.139245	4.356791	1.163778
**C**	–5.338499	–2.716326	–1.393015
**H**	–5.454831	–2.471157	–0.337362
**H**	–6.173605	–3.348249	–1.692165
**C**	3.176629	–1.915372	–3.261218
**H**	2.374801	–1.443043	–3.825769

**H**	3.919168	–2.285865	–3.969916
**C**	0.274768	–0.402901	–1.157794
**C**	–3.968011	–3.360098	–1.630426
**H**	–3.950806	–3.892388	–2.583910
**H**	–3.680821	–4.055227	–0.842401
**C**	2.680741	–3.053065	–2.361253
**H**	2.570547	–4.001071	–2.886129
**H**	1.726392	–2.779718	–1.911824
**C**	–3.063569	–2.136731	–1.680427
**H**	–2.790587	–1.813922	–0.671075
**H**	–2.157328	–2.272173	–2.269704
**C**	3.763684	–3.092287	–1.292709
**H**	3.425695	–3.503504	–0.340540
**H**	4.636580	–3.665100	–1.626634
**H**	–0.354822	–1.141830	–0.683910
**H**	0.598635	–0.596911	–2.171149
**H**	2.015249	3.870898	–1.122346
**C**	6.291638	0.926586	–0.159487
**H**	6.014671	–0.095575	–0.417576
**H**	7.243361	0.906845	0.378091
**C**	3.817127	–0.948452	–2.260772
**H**	4.733690	–0.494878	–2.641635
**H**	3.124190	–0.158291	–1.969172
**C**	0.030720	–2.561111	3.265323
**O**	1.037020	–2.766742	2.247739
**C**	0.481374	–3.544303	1.166822
**C**	–1.025867	–3.541390	1.384776
**C**	–1.128347	–3.487095	2.912203
**H**	0.478131	–2.774071	4.237000
**H**	–0.287030	–1.514629	3.241988
**H**	0.776273	–3.080511	0.224912
**H**	0.896174	–4.557135	1.212774
**H**	–1.472900	–2.641884	0.961379
**H**	–1.513392	–4.412477	0.948726
**H**	–2.084183	–3.098276	3.256596
**H**	–0.972334	–4.476826	3.346504

4.3 Data of the calculated monomeric and dimeric benzyl compounds

In the following, bond length of the calculated monomers and dimers (Table S14) are listed and the calculated dimers with the corresponding NBO-charge distribution of the different benzyl moieties are visualized. The visualization was done with the programs Molden and IboView.

Table S14 Bond lengths of the different benzyl fragments (i = isolated, s = sandwiched) in calculated monomeric and dimeric units as well as comparison with compounds **3a**, **3b**, **3c** and **3d**. Level of theory B3LYP-D3/def2-tzvpp.

Calculation number	M	C_α–C_i [Å]	C_i–C_o [Å]	C_o–C_m [Å]	C_m–C_p [Å]
**QM1**	Na	1.412	1.436<br>1.436	1.383<br>1.383	1.397<br>1.399
**QM2**	K	1.389	1.446<br>1.446	1.381<br>1.381	1.404<br>1.403
**QM3**	Rb	1.389	1.445<br>1.445	1.380<br>1.380	1.404<br>1.404
**QM4**	Cs	1.390	1.444<br>1.443	1.381<br>1.381	1.403<br>1.404
**QM5 (s)**	Na	1.398	1.438<br>1.441	1.390<br>1.378	1.404<br>1.395
**QM6 (s)**	K	1.389	1.444<br>1.444	1.383<br>1.379	1.407<br>1.401
**QM7 (s)**	Rb	1.388	1.447<br>1.445	1.381<br>1.384	1.402<br>1.405
**QM8 (s)**	Cs	1.391	1.445<br>1.442	1.381<br>1.385	1.399<br>1.403
**QM5 (i)**	Na	1.384	1.447<br>1.447	1.380<br>1.380	1.408<br>1.408
**QM6 (i)**	K	1.385	1.446<br>1.446	1.380<br>1.380	1.407<br>1.406
**QM7 (i)**	Rb	1.392	1.442<br>1.443	1.381<br>1.381	1.404<br>1.405
**QM8 (i)**	Cs	1.389	1.443<br>1.444	1.380<br>1.381	1.404<br>1.405
**3a**	Na	1.411(2)	1.436(2)<br>1.433(2)	1.384(2)<br>1.380(2)	1.397(2)<br>1.396(2)
**3b**	K	1.367(5)	1.437(5)<br>1.451(5)	1.372(5)<br>1.372(5)	1.379(5)<br>1.403(4)
**3c**	Rb	1.357(8)	1.442(9)<br>1.446(11)	1.335(11)<br>1.395(9)	1.362(14)<br>1.390(11)
**3d**	Cs	1.381(2)	1.445(2)<br>1.442(2)	1.379(3)<br>1.378(2)	1.401(2)<br>1.401(2)

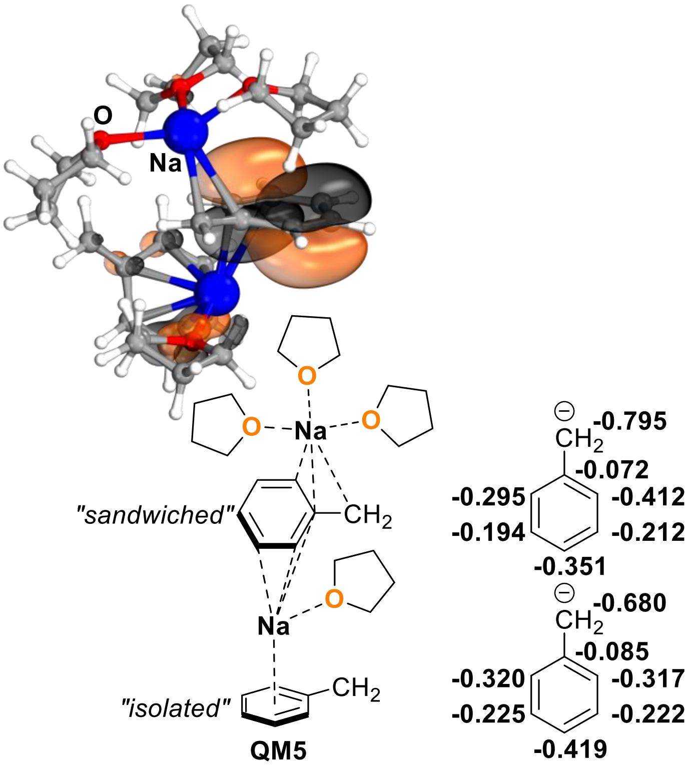

Figure S19 Visualization of QM5 and the HOMO-4 with the “isolated” and “sandwiched” fragment and representation of the NBO-charges at comparable carbon centers of the benzyl anion.

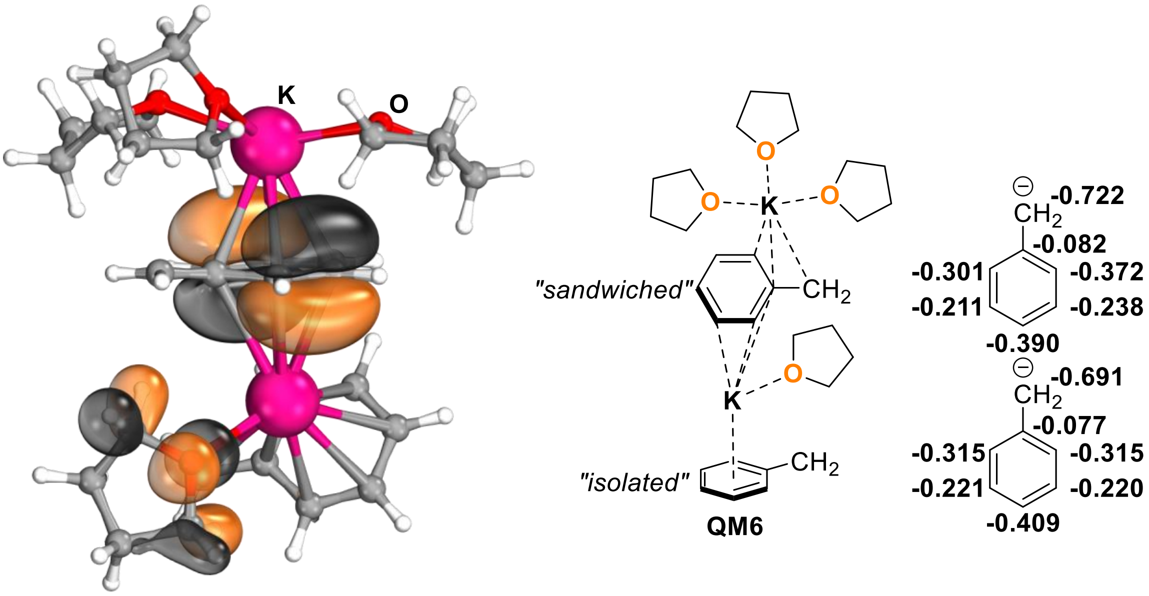

Figure S20 Visualization of QM6 and the HOMO-4 with the “isolated” and “sandwiched” fragment and representation of the NBO-charges at comparable carbon centers of the benzyl anion.

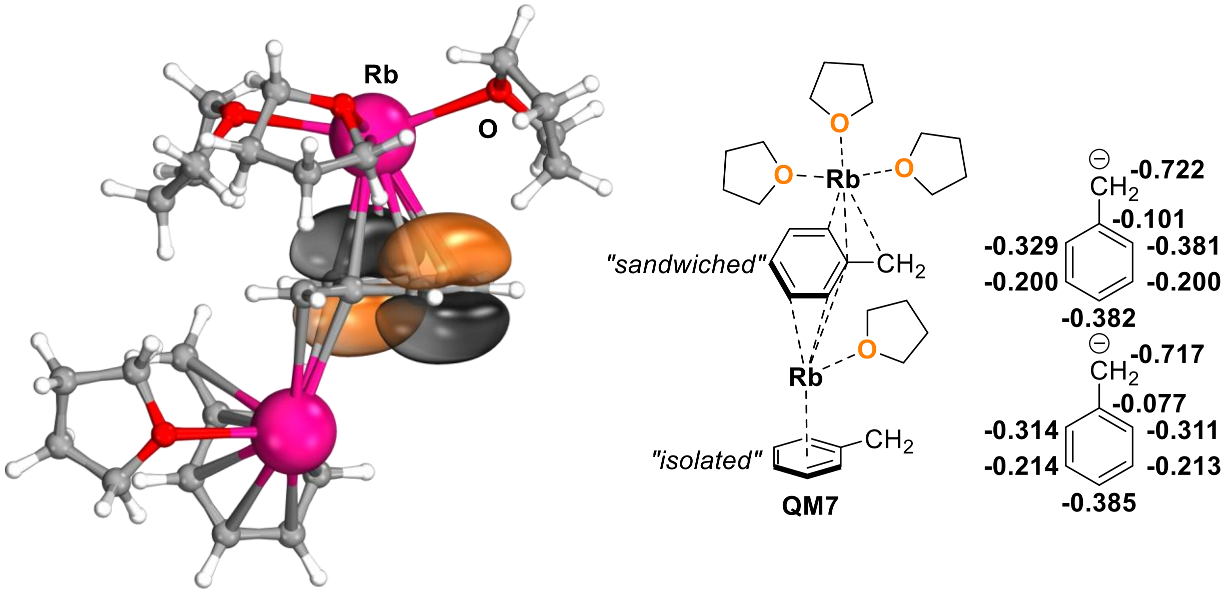

Figure S21 Visualization of QM7 and the HOMO-4 with the "isolated" and "sandwiched" fragment and representation of the NBO-charges at comparable carbon centers of the benzyl anion.

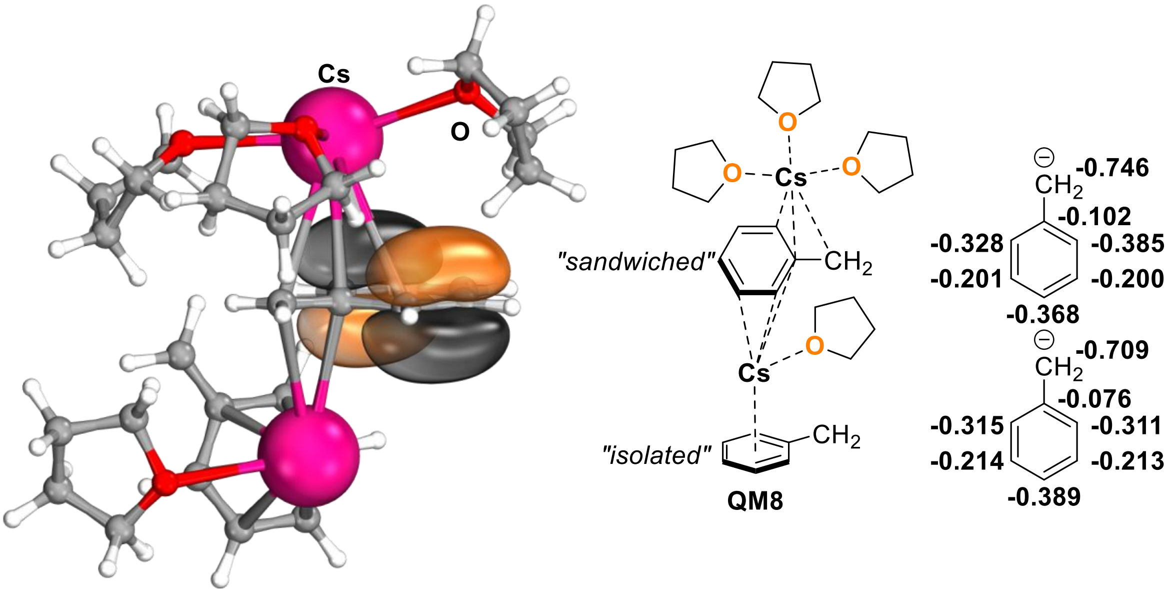

Figure S22 Visualization of QM8 and the HOMO-4 with the "isolated" and "sandwiched" fragment and representation of the NBO-charges at comparable carbon centers of the benzyl anion.

4.3.1 Summary

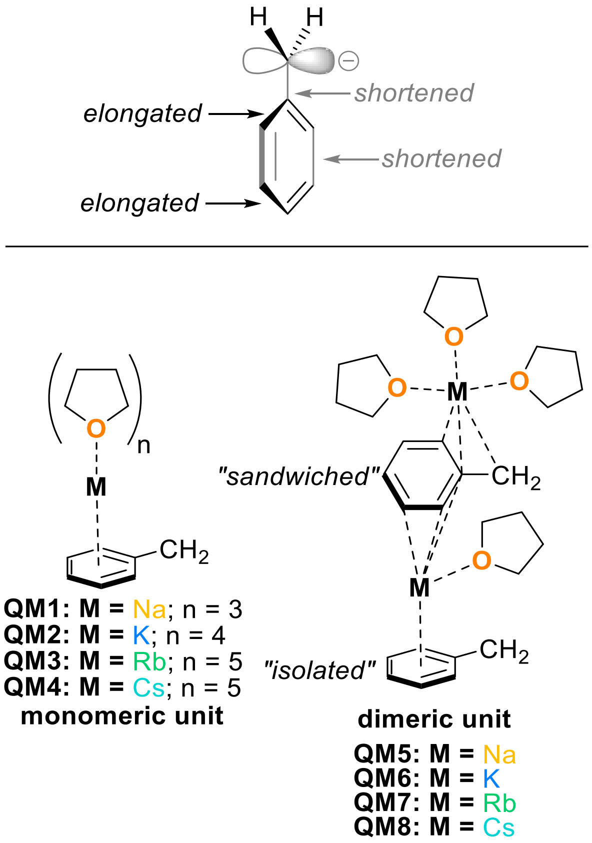

Figure S23 Trends of the bond length changes in the benzyl anion as well as the monomeric and dimeric units for the quantum chemical calculations.

A comparison of the calculated monomeric units illustrates a change in the bond lengths of the anion depending on the metal. In all calculations, a charge delocalization is recognizable due to the change in bond lengths. In contrast to K–Cs (**QM2-QM4)** the bond shortenings and elongations are not quite as pronounced for **QM1**. For t **QM2-QM4**, the charge in the benzyl anion is much more delocalized. Therefore, we obtain substantial shortening and elongation of the corresponding bonds. The charge delocalization appears to be very symmetrical in the monomeric units as the bond length of C_{ipso}–C_{ortho}, C_{ortho}–C_{meta} and C_{meta}–C_{para} are very similar to each other (see ESI). The dimeric unit contains two different benzyl anions, which are "sandwiched" by two metals on the one hand and "isolated" on the other. The "sandwiched" benzyl fragment features the same trends for the bond length changes as the calculated monomeric units, whereas the trend in the isolated fragment is clearly different. Surprisingly, the sodium compound **QM5** reveals the shortest C_{α}–C_{ipso} bond for the "isolated" fragment as it forms a η^6-contact with the isolated benzyl moiety, forcing the negative charge to delocalize and increase the Coulomb interaction with the metal. As expected, calculations of the heavier alkali metals K–Cs (**QM6-QM8**) stay in the same range. The sandwiched benzyl unit shows a conspicuous feature, as the charge distribution appears to be asymmetric. Comparable bond lengths differ clearly because both, the alkali metal above and underneath the sandwiched fragment are not located centrally above the phenyl ring but shifted to one side. For example, the NBO-charges of the comparable C_{ortho}-centers of the sandwich benzyl moiety differ significantly because only one side of the fragment interacts with both metals. The results of the quantum chemical calculations are consistent

with the experimentally obtained results. On the one hand, the benzylsodium thf solvate **3a** shows a symmetrical structure in the solid state with both metals, above and underneath, forming contacts to the benzyl unit in the same manner. Accordingly, the charge is symmetrically distributed over the phenyl ring, which is reflected in the bond lengths. These results agree best with the calculated bond lengths in the monomeric unit or the isolated benzyl moiety in the dimer. For the benzylpotassium thf solvate **3b** and the benzylrubidium thf solvate **3c**, on the other hand, an asymmetric charge distribution, illustrated by the bond lengths, can be observed. The calculated model that comes closest to this observation is the dimeric unit with the sandwiched benzyl moiety since it reflects the asymmetric charge distribution. The experimental data of the benzylcaesium thf solvate **3d** reveal a symmetric delocalization of the negative charge as comparable bond length does not differ from each other. However, the coordination sphere of the benzyl units is very different from the other examples, since each anion is surrounded by four metal centers. Calculations of a monomeric benzylcaesium species fit best to the experimentally obtained results, although the coordination sphere of benzyl moiety is not quite correctly represented. In summary, these quantum chemical calculations elucidate the influence of the coordination environment on the charge distribution of the benzyl anions. A symmetrical coordination sphere leads to a symmetrical delocalization of the negative charge, the opposite can be observed with an asymmetrical sphere. As a result, the coordination modes of the specific metals found in the solid are best represented by the monomeric model (Na, Cs) on the one hand and by the dimeric model (K, Rb) on the other hand, which is reflected in the charge distributions of the anion.

4.4 Isodesmic reaction towards the deprotonation of *N*,*N*-Dimethyl-2,2-diphenylethan-1-amine (XX)

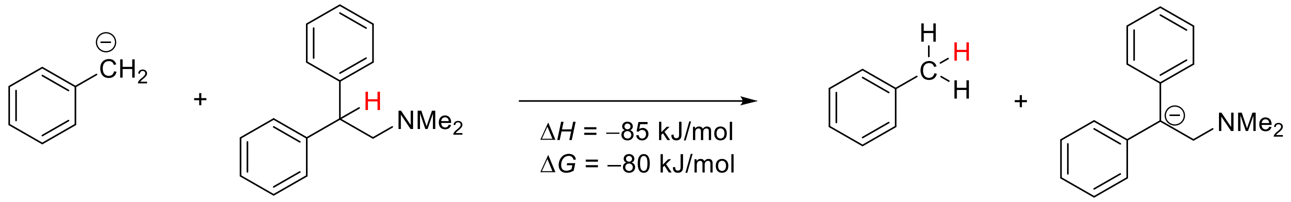

QM9 **4/QM10** **1/QM11** **QM12**

Scheme S1 Isodesmic reaction towards the stability of the anion **QM10**.

Table S15 Total (SCF) and zero-point-corrected (ZPE) energies of the optimized structures used in the isodesmic reaction.

Name	Calculation number	SCF [Hartree]	ZPE [Hartree]
**Benzylanion**	QM9	–270.820930890	–270.707497
**4**	QM10	–675.595613947	–675.280756
**1**	QM11	–271.441449922	–271.312248
**Amineanion**	QM12	–675.008261877	–674.708296

Table S16 Illustration of product stability by thermodynamic quantities.

	ΔZPE [kJ·mol^{-1}]	Δ*H* [kJ·mol^{-1}]	Δ*G* [kJ·mol^{-1}]
**Sum of products –sum of educts**	–84.78	–85.27	–80.70

Table S17 Coordinates of **QM9**.

Element	X	Y	Z
**C**	–0.194957	1.200673	–0.009237
**C**	1.198854	1.203754	0.002137
**C**	1.901213	–0.000190	0.008539
**C**	1.198398	–1.204009	0.002139
**C**	–0.195279	–1.200472	–0.009239
**C**	–0.911733	0.000277	–0.012085
**H**	–0.736315	2.144245	–0.017922
**H**	1.736486	2.147762	0.002243
**H**	2.987214	–0.000403	0.014434
**H**	1.735782	–2.148160	0.002243
**H**	–0.737017	–2.143849	–0.017933
**C**	–2.420177	0.000081	0.009989
**H**	–2.824684	–0.880172	–0.498307
**H**	–2.794741	–0.011625	1.040166
**H**	–2.824642	0.891512	–0.478381

Table S 18 Coordinates of **4/QM10**.

Element	X	Y	Z
**C**	3.786916	–0.182861	–0.222269
**C**	2.468301	–0.609904	–0.127887
**C**	1.389649	0.270048	0.195557
**C**	1.791684	1.621377	0.431259
**C**	3.109755	2.040920	0.330263
**C**	4.138359	1.152282	–0.006146
**H**	4.555159	–0.908907	–0.486409
**H**	2.253202	–1.654149	–0.334883
**H**	1.043408	2.336639	0.757642
**H**	3.344720	3.083786	0.540909
**H**	5.169017	1.486222	–0.089611
**C**	0.032147	–0.191256	0.334151
**C**	–0.194412	–1.603291	0.790885
**H**	–0.820979	–1.619658	1.700259
**H**	0.765296	–2.067949	1.087965
**C**	–1.127001	0.633993	0.151866
**C**	–1.112635	1.903872	–0.508096
**C**	–2.432468	0.198373	0.546245
**C**	–2.252875	2.675740	–0.674510
**H**	–0.182097	2.264579	–0.933805
**C**	–3.564622	0.985141	0.384617
**H**	–2.556674	–0.795571	0.962270
**C**	–3.503315	2.248073	–0.212680
**H**	–2.165381	3.630731	–1.192019
**H**	–4.524099	0.593972	0.722144
**H**	–4.393271	2.858452	–0.340222
**N**	–0.891181	–2.489289	–0.168353
**C**	–1.123411	–3.790822	0.407451
**H**	–1.700672	–3.689789	1.334252
**H**	–1.694684	–4.417092	–0.288501
**H**	–0.178082	–4.324621	0.651435
**C**	–0.185219	–2.598005	–1.425769
**H**	0.769520	–3.158136	–1.327602
**H**	–0.810508	–3.122170	–2.159016
**H**	0.039280	–1.593400	–1.795947

Table S19 Coordinates of **1/QM11**.

Element	X	Y	Z
**C**	0.243775	–1.210182	–0.000027
**C**	–1.137985	–1.197466	–0.000023
**C**	–1.877961	0.000000	–0.000002
**C**	–1.137985	1.197466	0.000008
**C**	0.243775	1.210182	0.000008
**C**	1.038331	–0.000000	0.000007

H	0.770652	–2.164708	–0.000051
H	–1.667847	–2.151932	–0.000045
H	–2.964312	0.000000	0.000010
H	–1.667847	2.151932	0.000015
H	0.770653	2.164708	0.000011
C	2.425265	0.000000	0.000026
H	2.987707	0.930185	0.000052
H	2.987707	–0.930184	0.000030

Table S20 Coordinates of **QM12**.

Element	X	Y	Z
C	3.552531	–0.609444	1.058781
C	2.175130	–0.787596	0.913051
C	1.512840	–0.339863	–0.232884
C	2.265909	0.293903	–1.228582
C	3.638226	0.473514	–1.089016
C	4.288662	0.020234	0.059627
H	4.047506	–0.965874	1.957781
H	1.622033	–1.280734	1.707622
H	1.759868	0.657224	–2.121066
H	4.201600	0.966565	–1.876135
H	5.360124	0.157157	0.172341
C	0.011736	–0.474332	–0.427685
H	–0.144226	–0.737640	–1.481700
C	–0.606668	–1.594103	0.430884
H	–0.624203	–1.258925	1.474878
H	0.035782	–2.496125	0.385144
C	–0.720310	0.838454	–0.182856
C	–1.865206	1.144421	–0.924190
C	–0.304475	1.734371	0.806365
C	–2.578221	2.317550	–0.686964
H	–2.204935	0.447531	–1.686461
C	–1.018593	2.906604	1.051073
H	0.592906	1.520618	1.382588
C	–2.157035	3.203219	0.303915
H	–3.464151	2.539174	–1.275425
H	–0.678644	3.592793	1.821728
H	–2.709790	4.119615	0.489661
N	–1.973666	–1.907287	0.041732
C	–2.695982	–2.548979	1.126440
H	–2.719288	–1.887593	1.996996
H	–3.727161	–2.741285	0.815533
H	–2.240647	–3.512750	1.426776
C	–2.024437	–2.731334	–1.153344
H	–1.545778	–3.718276	–1.001103
H	–3.067610	–2.894187	–1.440035
H	–1.522362	–2.235983	–1.988942

5. References

[1] T. Kottke, D. Stalke, *J. Appl. Cryst.* **1993**, *26*, 615–619.
[2] O. V. Dolomanov, L. J. Bourhis, R. J. Gildea, J. A. K. Howard, H. Puschmann, *J. Appl. Cryst.* **2009**, *42*, 339–341.
[3] a) G. M. Sheldrick, *Acta Cryst.* **2015**, *A71*, 3–8; b) G. M. Sheldrick, *Acta Cryst.* **2008**, *A64*, 112–122.
[4] G. M. Sheldrick, *Acta Cryst.* **2015**, *C71*, 3–8.
[5] a) C. Unkelbach, D. F. O'Shea, C. Strohmann, *Angew. Chem. Int. Ed.* **2014**, *53*, 553–556; b) C. Unkelbach, D. F. O'Shea, C. Strohmann, *Angew. Chem.* **2014**, *126*, 563–567.
[6] a) A. Seymen, U. Opper, A. Voß, L. Brieger, F. Otte, C. Unkelbach, D. F. O'Shea, C. Strohmann, *Angew. Chem. Int. Ed.* **2020**, *59*, 22500–22504; b) A. Seymen, U. Opper, A. Voß, L. Brieger, F. Otte, C. Unkelbach, D. F. O'Shea, C. Strohmann, *Angew. Chem.* **2020**, *132*, 22688–22693.
[7] L. J. Farrugia, *J. Appl. Cryst.* **1997**, *30*, 565.
[8] M. J. Frisch, G. W. Trucks, H. B. Schlegel, G. E. Scuseria, M. A. Robb, J. R. Cheeseman, G. Scalmani, V. Barone, G. A. Petersson, H. Nakatsuji, X. Li, M. Caricato, A. V. Marenich, J. Bloino, B. G. Janesko, R. Gomperts, B. Mennucci, H. P. Hratchian, J. V. Ortiz, A. F. Izmaylov, J. L. Sonnenberg, D. Williams-Young, F. Ding, F. Lipparini, F. Egidi, J. Goings, B. Peng, A. Petrone, T. Henderson, D. Ranasinghe, V. G. Zakrzewski, J. Gao, N. Rega, G. Zheng, W. Liang, M. Hada, M. Ehara, K. Toyota, R. Fukuda, J. Hasegawa, M. Ishida, T. Nakajima, Y. Honda, O. Kitao, H. Nakai, T. Vreven, K. Throssell, J. A. Montgomery, Jr., J. E. Peralta, F. Ogliaro, M. J. Bearpark, J. J. Heyd, E. N. Brothers, K. N. Kudin, V. N. Staroverov, T. A. Keith, R. Kobayashi, J. Normand, K. Raghavachari, A. P. Rendell, J. C. Burant, S. S. Iyengar, J. Tomasi, M. Cossi, J. M. Millam, M. Klene, C. Adamo, R. Cammi, J. W. Ochterski, R. L. Martin, K. Morokuma, O. Farkas, J. B. Foresman, and D. J. Fox, *Gaussian 16*, Gaussian Inc., Wallingford CT.
[9] a) C. Lee, W. Yang, R. G. Parr, *Phys. Rev. B* **1988**, *37*, 785–789; b) S. Grimme, J. Antony, S. Ehrlich, H. Krieg, *J. Chem. Phys.* **2010**, *132*, 154104.
[10] F. Weigend, R. Ahlrichs, *Phy. Chem. Chem. Phys.* **2005**, *7*, 3297–3305.
[11] a) P. Hohenberg, W. Kohn, *Phys. Rev.* **1964**, *136*, B864-B871; b) W. Kohn, L. J. Sham, *Phys. Rev.* **1965**, *140*, A1133-A1138.
[12] Roy Dennington, T. A. Keith, J. M. Millam, *GaussView, Version 6.0*, Semichem Inc., Shawnee Misssion, KS, **2016**.
[13] a) J. P. Foster, F. Weinhold, *J. Am. Chem. Soc.* **1980**, *102*, 7211–7218; b) A. E. Reed, F. Weinhold, *J. Chem. Phys.* **1983**, *78*, 4066–4073; c) A. E. Reed, F. Weinhold, *J. Chem. Phys.* **1985**, *83*, 1736–1740; d) A. E. Reed, R. B. Weinstock, F. Weinhold, *J. Chem. Phys.* **1985**, *83*, 735–746; e) A. E. Reed, L. A. Curtiss, F. Weinhold, *Chem. Rev.* **1988**, *88*, 899–926.
[14] G. Schaftenaar, J. H. Noordik, *J. Comput. Aided Mol. Des.* **2000**, *14*, 123–134.
[15] a) G. Knizia, *J. Chem. Theory Comput.* **2013**, *9*, 4834–4843; b) G. Knizia, J. E. M. N. Klein, *Angew. Chem. Int. Ed.* **2015**, *54*, 5518–5522.
